# Constraining effects of aerosol-cloud interaction by accounting for coupling between cloud and land surface

**DOI:** 10.1126/sciadv.adl5044

**Published:** 2024-05-23

**Authors:** Tianning Su, Zhanqing Li, Natalia Roldan Henao, Qingzu Luan, Fangqun Yu

**Affiliations:** ^1^ Earth System Science Interdisciplinary Center & AOSC, University of Maryland, College Park, MD, USA.; ^2^ Atmospheric Sciences Research Center, University at Albany, Albany, NY, USA.

## Abstract

Aerosol-cloud interactions (ACIs) are vital for regulating Earth’s climate by influencing energy and water cycles. Yet, effects of ACI bear large uncertainties, evidenced by systematic discrepancies between observed and modeled estimates. This study quantifies a major bias in ACI determinations, stemming from conventional surface or space measurements that fail to capture aerosol at the cloud level unless the cloud is coupled with land surface. We introduce an advanced approach to determine radiative forcing of ACI by accounting for cloud-surface coupling. By integrating field observations, satellite data, and model simulations, this approach reveals a drastic alteration in aerosol vertical transport and ACI effects caused by cloud coupling. In coupled regimes, aerosols enhance cloud droplet number concentration across the boundary layer more homogeneously than in decoupled conditions, under which aerosols from the free atmosphere predominantly affect cloud properties, leading to marked cooling effects. Our findings spotlight cloud-surface coupling as a key factor for ACI quantification, hinting at potential underassessments in traditional estimates.

## Teaser

Accounting for cloud-surface coupling refines aerosol-cloud interaction estimations, altering the magnitude of their effects.

## INTRODUCTION

Aerosol-cloud interactions (ACIs) have been recognized as playing the central role in the regulation of the energy balance and climate of the Earth (*1*–*5*). By serving as cloud condensation nuclei (CCNs), aerosols can regulate cloud properties and the hydrologic cycle and, thus, they exert important forcing over the radiation budget and climate change (*6*–*13*). However, the quantification of ACI, particularly regarding the magnitude of radiative forcing by ACIs (RF_aci_), remains highly uncertain, with substantial discrepancies persisting between observational-based estimates and modeled values (*1*, *14*–*16*). In general, the community tends to rely on the observation-based estimates of RF_aci_ with a global mean ranging from −0.2 to −1.0 W m^−2^ (*1*, *16*–*18*), lower than the modeled range of −0.3 to −1.8 W m^−2^ (*2*, *19*). Reconciling these differences is essential to improve the estimation of the ACI, by means of both observational analysis and model simulation (*20*, *21*).

An important source of uncertainty in the quantification of RF_aci_ lies in the difficulty of the direct measurement of concentrations of CCNs at cloud base (*22*–*26*). Because measurements of CCN at the cloud level are scarce, various CCN proxy variables have been proposed and used using more conventional measurements (*17*, *27*–*29*), including surface sulfate aerosols (*18*), aerosol optical depth (AOD) (*16*, *17*, *30*, *31*), and aerosol extinction (*32*, *33*) from which attempts were made to retrieve the CCN (*34*, *35*), but their effectiveness in a wide range of conditions remains uncertain. This issue is particularly challenged by the inadequate vertical information of aerosol proxy measurements.

Previous studies have underscored the importance of aligning aerosol and cloud layers vertically for investigating the relationship between cloud microphysics and aerosol properties (*22*, *36*, *37*). Costantino and Bréon (*38*, *39*) found that the microphysical parameters of clouds align more accurately with aerosol properties when vertical alignment is considered. Similarly, Painemal *et al.* (*32*) observed a stronger correlation between cloud droplet number concentration (*N*_d_) and aerosol extinction coefficients near clouds from space-borne lidar than with AOD. Meanwhile, the *N*_d_-CCN relationship weakens as the planetary boundary layer (PBL) deepens, suggesting that surface aerosol measurements may not effectively represent aerosol variability at the cloud base in thicker PBL (*40*). This issue was critically reviewed and summarized by Quaas *et al.* (*37*), noting that the lack of vertical alignment between CCN proxies and clouds leads to an underestimation of *N*_d_-CCN sensitivity, further aggravated by the availability and uncertainties in the retrievals of vertical profiles of aerosol and CCNs.

Therefore, there is notable scope to refine ACI quantification by addressing the uncertainties arising from variable aerosol vertical distributions beneath clouds. We hypothesize that this challenge is intrinsically tied to cloud-surface coupling processes, which are deeply intertwined with boundary layer processes (*41*). Cloud-surface coupling refers to the exchange of turbulent fluxes between the surface and cloud through the PBL (*42*, *43*). Given that most aerosols reside within the PBL, cloud-surface coupling notably influences aerosol transport from the boundary layer to the cloud base (*44*) and, in turn, ACI by modulating aerosol vertical distribution in the subcloud layer. While the influence of the underlying surface on ACI has been recognized (*29*, *45*), quantifying RF_aci_ while considering the states of cloud-land-surface coupling remains underexplored. The recent development of a methodology to determine cloud-surface coupling (*46*) lays the groundwork for investigating this issue comprehensively.

In this study, we aim to illuminate the influence of cloud-surface coupling on aerosol structure and quantify its resulting changes in ACI estimates. This approach diverges from existing methods that depend on lidar retrievals for assessing aerosol vertical distributions, which are limited by signal noise, cloud contamination, and sampling constraints. By integrating comprehensive observations, we examine the roles of cloud-surface coupling in aerosol transport and its impacts on the cloud properties, in particular *N*_d_ and RF_aci_ across different coupling scenarios. This comprehensive analysis leads to a framework in the estimation of RF_aci_, with a notable advancement in narrowing down the effects of land surface coupling.

## RESULTS

### Impacts of cloud-surface coupling on aerosol vertical distributions

**Fig. 1.**
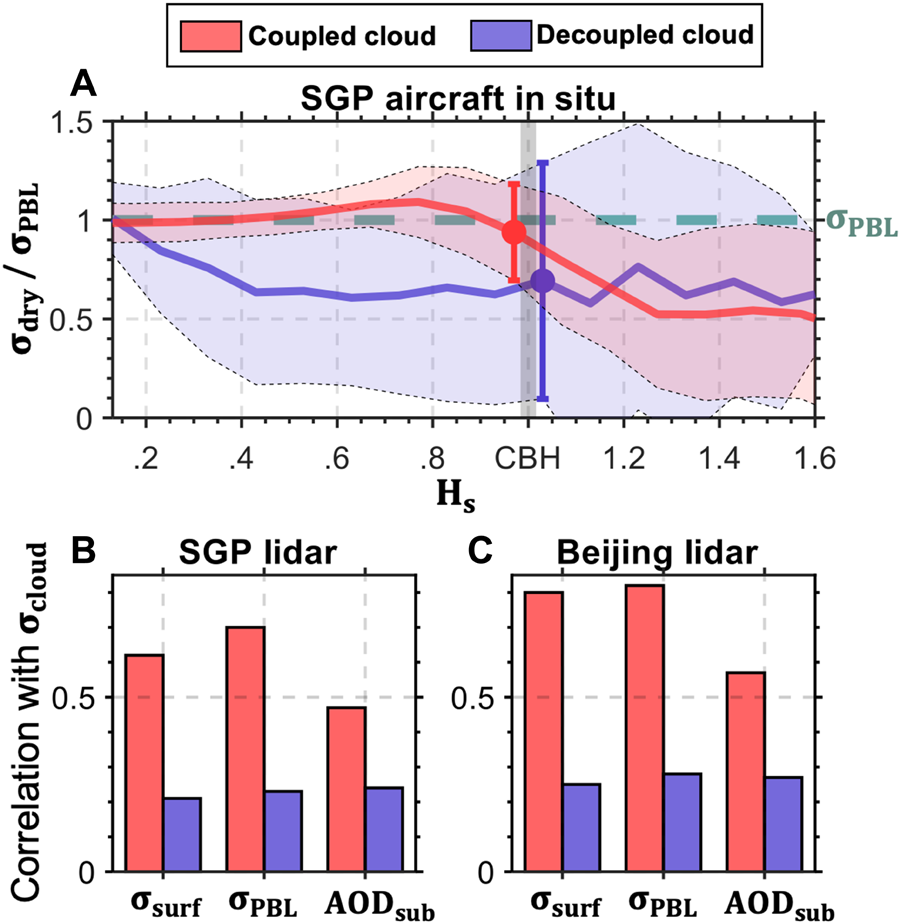
Subcloud aerosol variations under coupled and decoupled regimes. (**A**) Normalized aerosol extinction profiles measured by aircraft in situ within 15-km around the Southern Great Plains (SGP) site. Solid lines represent the ratio of dry fine-mode aerosol extinction (σ_dry_) to mean aerosol extinction within the boundary layer (σ_PBL_) as a function of normalized height (*H*_s_), where *H*_s_ is defined as the height divided by the cloud base height (CBH). The shaded areas indicate the standard deviation of σ_dry_/σ_PBL_, with different colors representing coupled (red) and decoupled (blue) cloud conditions. The error bars represent the stand deviations of σ_dry_/σ_PBL_ near the cloud base. (**B**) Correlation coefficients between aerosol extinction at the CBH (σ_cloud_) and different aerosol indexes, including near-surface aerosol extinction (σ_surf_), σ_PBL_, and AOD_sub_ derived from ground-based lidar measurements over (B) the SGP and (**C**) Beijing. σ_cloud_ is computed as the mean extinction from neighboring clear pixels at the CBH.

To understand the influence of cloud-surface coupling on aerosol vertical distributions, we investigated subcloud aerosol variations under different cloud-surface coupling conditions. Figure 1A, using data from the in situ aerosol profiles (IAP) campaign that conducted over 600 flights from 2000 to 2006, presents the ratio of dry fine-mode aerosol extinction (σ_dry_) to mean aerosol extinction within the boundary layer (σ_PBL_). This analysis illustrates aerosol vertical variabilities under coupled and decoupled cloud conditions. The spatial and vertical frequencies of in situ aerosol measurements are presented in fig. S1 (dataset described in Materials and Methods). These observations reveal distinct subcloud aerosol variations under coupled and decoupled regimes.

In the case of a coupled cloud, turbulence originating from the surface can extend to the cloud base, influencing cloud evolution and creating a turbulent linkage among surface, the PBL, and the cloud. In the absence of this interaction, the cloud is considered to be in a decoupled scenario. The vertical distribution of aerosol loading beneath cloud (hereafter subcloud) exhibits distinct differences between the coupled and decoupled regimes (two cases demonstrated in figs. S2 and S3). In the coupled regime, aerosols generally mix well throughout the subcloud layer, evidenced by the consistent aerosol profile with smaller variabilities in Fig. 1A. Conversely, in the decoupled regime, aerosol distribution becomes heterogeneous, with a marked difference in aerosol concentrations between surface and cloud base. Weaker vertical mixing in the decoupled regime, which limits the upward transport of aerosols, accounts for this phenomenon, leading to aerosol accumulation near the surface.

In addition to the in situ aircraft data, this study incorporates aerosol extinction profiles from ground-based lidars at the southern Great Plains (SGP) and Beijing sites, selecting data segments free of cloud contamination. These profiles help illustrate the consistency between σ_PBL_, σ_cloud_ (aerosol extinction at the cloud base), and AOD_sub_ (subcloud AOD) in coupled conditions, as well as the differences observed in decoupled scenarios (Fig. 1, B and C). We also ruled out the possibility that different cloud bases notably contribute to the difference in aerosol concentrations between coupled and decoupled regimes (fig. S4). These findings underscore the substantial influence of the cloud coupling state on the aerosol vertical distribution, holding implications for quantifying ACI.

### Responses of clouds to aerosols

**Fig. 2.**
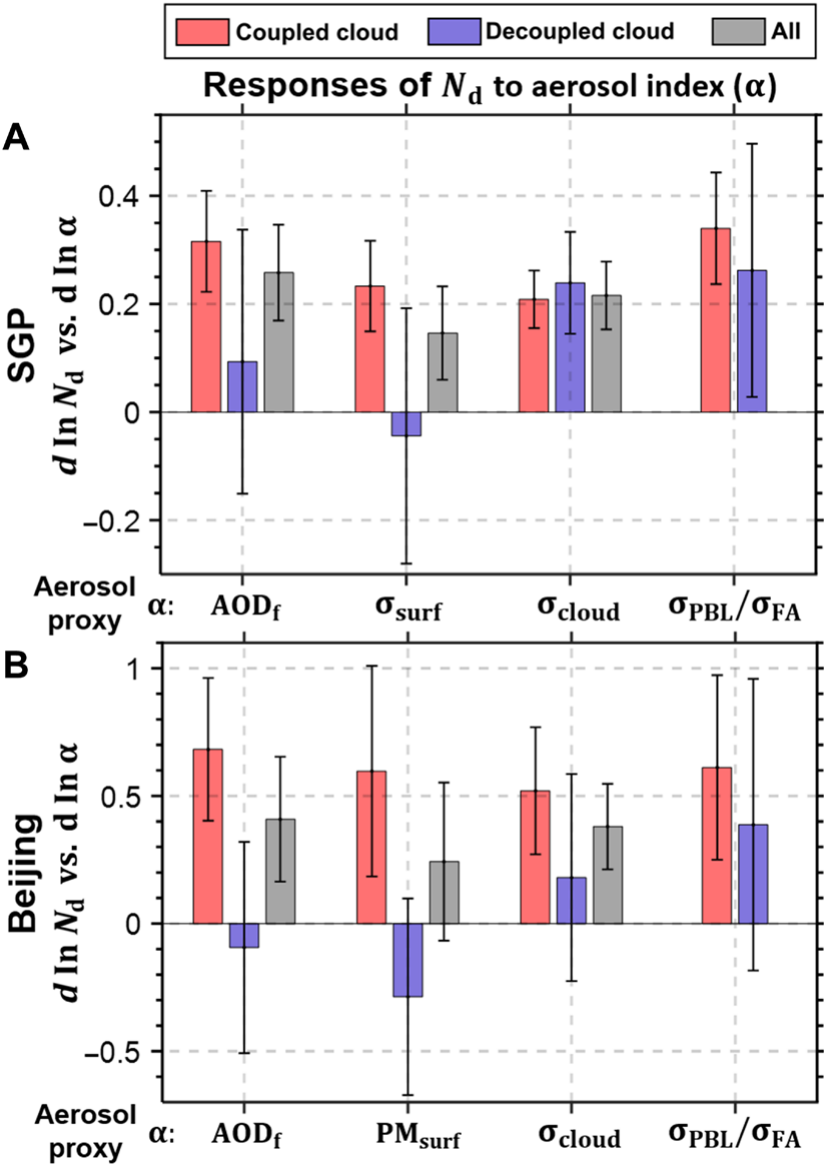
The responses of cloud droplet number concentration to aerosol under different coupling regimes. The responses $\left(\frac{d\text{ln}N_{d}}{d\text{ln}\alpha }\right)$ are calculated as the slopes of linear regression between *d*ln*N*_d_ and *d*lnα for liquid water clouds over (**A**) the SGP and (**B**) Beijing. Red, blue, and gray bars indicate the responses for coupled cases, decoupled cases, and all cases. The aerosol proxies used are fine-mode aerosol optical depth (AOD_f_) derived from MERRA-2, the surface fine-mode (diameter <1 μm) aerosol extinction (fine-mode σ_surf_), surface PM_2.5_ derived from in situ measurements, aerosol extinction below clouds derived from lidar (σ_cloud_), and mean σ_dry_ within PBL (σ_PBL_) and mean σ_dry_ within free atmosphere (σ_FA_) derived from MERRA-2. σ_FA_ is calculated as the mean σ_dry_ between cloud base and 600 hPa. The error bars indicate the 90% confidence level.

Preceding observations suggest that substantial differences in the ACI under different states of cloud-surface coupling are rooted in different transport of aerosol from within the PBL, especially near the surface, to the cloud layer. To validate this hypothesis, we examined the influence of different aerosol proxies on cloud properties under both coupled and decoupled scenarios. As demonstrated in Fig. 2, we calculate the slopes of linear regression between the changes in *N*_d_ and the changes in aerosol proxies (*d*ln*N*_d_/*d*lnα), where α represents different aerosol proxies including fine-mode AOD (AOD_f_) derived from the Modern-Era Retrospective analysis for Research and Applications, version 2 (MERRA2) (*47*), the surface fine-mode aerosol extinction (diameter <1 μm) or surface particulate matter with diameters ≤2.5 μm (PM_2.5_) derived from in situ measurements. We also include aerosol extinction below clouds derived from lidar, and the mean σ_dry_ within the PBL (σ_PBL_) and the free atmosphere (σ_FA_) as derived from the MERRA2.

The sampling numbers for deriving the regressions in Fig. 2 are presented in fig. S5. *N*_d_ is calculated from cloud effective radius and cloud optical depth measured by the Moderate Resolution Imaging Spectroradiometer (MODIS) over Eastern Asia and the field observations over the SGP (see Materials and Methods). σ_FA_ is calculated as the mean σ_dry_ between the cloud-base and 600 hPa. In MERRA-2 data, conversions between aerosol mixing ratios, PM, and σ_dry_ were implemented. Despite these conversions, the responses of *N*_d_ to either PM or σ_dry_ exhibit notable similarity (fig. S6). Our observations indicate that the overall responses of *N*_d_ to different aerosol proxies exhibit a range from 0.15 to 0.26 over the SGP and from 0.24 to 0.41 over Beijing. These findings are in general alignment with regional averages reported in previous studies, such as 0.2 to 0.4 over Asia and 0.1 to 0.5 for the North America (*16*, *48*), albeit on the lower end for the latter. Furthermore, the responses of *N*_d_ to aerosols have a comparable value with global estimates of 0.2 to 0.4 as documented by Diamond *et al.* (*49*) and McCoy *et al.* (*18*).

Our analysis reveals that the responses of *N*_d_ to different aerosol proxies are notably different under coupled and decoupled conditions. Specifically, the *N*_d_ demonstrates substantial responses to surface aerosol loading and AOD_f_ under coupled conditions, while the responses are insignificant under decoupled conditions. In contrast, the response of *N*_d_ to aerosol extinction right beneath cloud is less sensitive to the state of cloud-surface coupling. These results highlight that the states of cloud-surface coupling notably shape the sensitivity of cloud properties to different aerosol proxies, especially those measured at the surface.

The findings of our study suggest that conventional aerosol proxies, such as AOD or surface aerosol loading, are suitable for coupled regimes since there is consistency between surface aerosols, boundary layer aerosols, and aerosol loading at the cloud base. However, they fail to represent the aerosol concentration in the cloud base under decoupled conditions. Lidar-derived aerosol extinction is helpful for decoupled cloud conditions. However, lidar-derived extinction suffers from great uncertainties due to severe cloud contamination.

As a remedy solution to the potential problem, this study introduces the use of σ_PBL_ and σ_FA_ as additional aerosol proxies. The boundary layer aerosol loading provides a better representation of the aerosol extinction at the cloud base under the coupled regime (Fig. 1 and fig. S7). Conversely, the free-atmosphere aerosols help gain insights into the aerosol conditions within the free atmosphere, above the boundary layer (fig. S7) and thus can be used as the aerosol proxy for the decoupled regime. These findings emphasize the importance of considering the state of cloud-surface coupling while selecting appropriate aerosol proxies for accurate ACI quantification.

### RF_aci_ under coupled and decoupled conditions

**Fig. 3.**
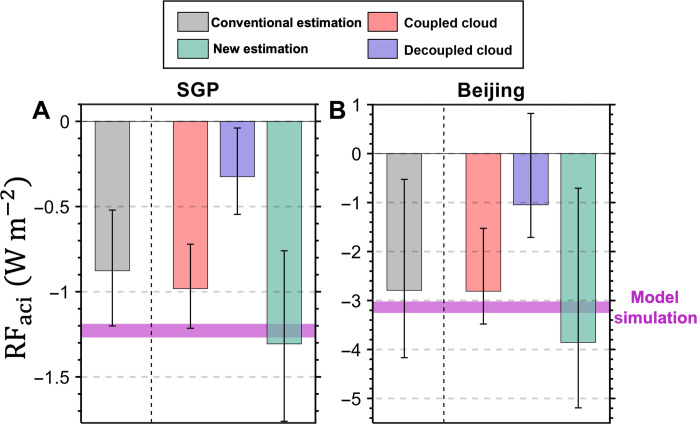
Comparative RF_aci_ under different coupling regimes. The gray bars indicate the averaged estimation (traditional estimation) of RF_aci_ by using fine-mode aerosol optical depth (AOD_f_) and surface aerosol loading as the aerosol proxies for (**A**) the Southern Great Plains (SGP) and (**B**) Beijing. We also separately consider the coupled (red bars) and decoupled (blue bars) regimes. In the coupled regime, mean σ_dry_ within PBL (σ_PBL_) is used as the aerosol proxy, while mean σ_dry_ within free atmosphere (σ_FA_) is used as the aerosol proxy in the decoupled regime. The green bars indicate the consolidated new estimation. The error bars indicate the 90% confidence level. For comparison, the pink line indicates the value of RF_aci_ from the GEOS-Chem model (*19*).

By using comprehensive field observations, we present the RF_aci_ in Fig. 3 (see Materials and Methods). The averaged estimation of RF_aci_ by using AOD_f_ and surface aerosol loading as the aerosol proxies is shown by the gray bars in Fig. 3 (referred as the traditional estimation). Compared to the model simulation from GEOS-Chem (*19*), the traditional observational estimation of RF_aci_ has a lower magnitude, which was the case in previous studies (*15*, *16*, *25*). The observed discrepancies between model outputs and observational estimates could originate from various sources, e.g., variations in spatial and temporal resolutions of the datasets, temporal and vertical variations in aerosol composition, and limitations in the model’s parameterization of ACIs. The systematic discrepancies are likely rooted, at least partially, to the representativeness of aerosol measurements for cloud base CCN.

To quantify this, we further differentiate RF_aci_ for coupled and decoupled regimes in our analyses. As RF_aci_ is delegated to represent the mean state at the top of atmosphere across all scenarios, the calculation of RF_aci_ accounts for the relative frequencies of coupled and decoupled clouds (see Materials and Methods). As mentioned in the previous section, we use σ_PBL_ and σ_FA_ as the aerosol proxies for coupled and decoupled regimes, respectively. The disparity in RF_aci_ between coupled and decoupled clouds is closely related to variations in their occurrence frequency and the sensitivity of *N*_d_ to aerosols. By deploying the revised aerosol proxies (σ_pbl_ and σ_FA_), the sensitivity of *N*_d_ to aerosols becomes relatively comparable between coupled and decoupled conditions (Fig. 2), while the higher prevalence of coupled clouds leads to a much higher value of RF_aci_ under the coupled conditions. Specifically, the difference in cloud frequency contributes to a decrease in RF_aci_ under decoupled conditions by 58% at SGP and 47% at Beijing compared to the coupled regime. The percentages of coupled and decoupled clouds at different cloud base heights (CBHs) are presented in fig. S8. In addition, the reduced sensitivity of *N*_d_ to aerosol under decoupled conditions further lowers RF_aci_ by 20% at SGP and 24% at Beijing, indicating a compound effect that intensifies the discrepancy in RF_aci_ between coupled and decoupled scenarios.

We also combined the new estimation of RF_aci_ as the aggregate of contributions from both coupled and decoupled cloud conditions, as indicated by the green bars in Fig. 3. The combined estimation of RF_aci_ is notably higher than the traditional estimation, highlighting the importance of selecting appropriate aerosol proxies according to the state of cloud-surface coupling. The results emphasize the necessity of considering the cloud-surface coupling state for accurate observational-based quantification of radiative forcing instigated by ACIs.

**Fig. 4.**
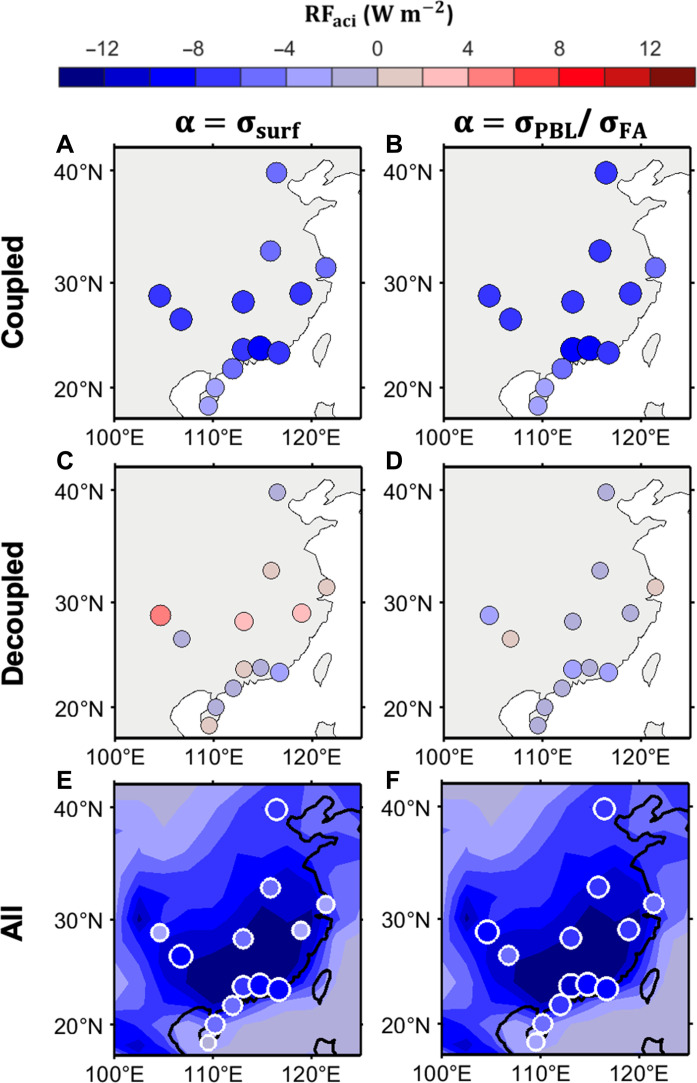
Summer radiative RF_aci_ over Eastern Asia. The dots are color-coded based on the RF_aci_ calculated from satellite estimations over radiosonde sites during summer for (**A** and **B**) coupled condition, (**C** and **D**) decoupled condition, and (**E** and **F**) all. The size of dots is adjusted according to the magnitude. The color shaded area in (E) and (F) represents the summer values of model simulations from the GEOS-Chem model (*19*). In (A), (C), and (E), we use surface level σ_dry_ as the aerosol proxy. In (B), (D), and (F), we use mean σ_dry_ within PBL (σ_PBL_) and mean σ_dry_ within free atmosphere (σ_FA_) as aerosol proxies for coupled and decoupled regimes, respectively. The state of cloud-surface coupling is diagnosed by radiosonde data. Observational RF_aci_ is calculated during the summertime of 2015–2019 due to the availability of noontime radiosonde.

Figure 4 shows the spatial distribution of RF_aci_ during summer over Eastern Asia for coupled and decoupled regimes, along with an overall case scenario, computed using satellite and radiosonde observational data (depicted by color dots). The state of cloud-surface coupling is determined using radiosonde data. The figure presents substantial differences in RF_aci_ between coupled and decoupled regimes across different sites, with coupled conditions exhibiting higher magnitudes than decoupled conditions. Consistent with the results in Beijing and SGP as illustrated in Fig. 3, the prominent disparities in RF_aci_ between coupled and decoupled regimes are largely attributed to the differences in their occurrence frequencies. Specifically, variations in cloud frequency contribute to an average reduction in RF_aci_ for decoupled conditions by 54%, with an 8% SD, across various sites. Meanwhile, our analysis indicates that upon applying aerosol proxies of σ_PBL_ and σ_FA_, the discrepancies in the sensitivity of *N*_d_ to aerosols between coupled and decoupled regimes became notably reduced (fig. S9).

In comparison, the RF_aci_ values from model simulations from the GEOS-Chem model are also portrayed by the shaded area for all cases only (Fig. 4, E and F). When using the traditional aerosol proxy, observational estimates of RF_aci_ tend to be lower than those simulated by the model, as is clear in Fig. 4E. However, using σ_PBL_ and σ_FA_ as aerosol proxies collectively account for the effects of cloud-surface coupling, making the gap between observational estimates and model simulations becomes smaller. The uncertainty that arises from the use of traditional aerosol proxies is thus reduced. This aspect is important as ACI can be modulated by the state of cloud-surface coupling. Owing to the enhanced vertical mixing within PBL, boundary layer aerosols transported to the cloud layer can effectively modulate cloud properties. In contrast, for the decoupled regime, vertical mixing in the subcloud layer was suppressed, hindering the effective transport of energy, aerosol, and moisture aloft.

## DISCUSSION

**Fig. 5.**
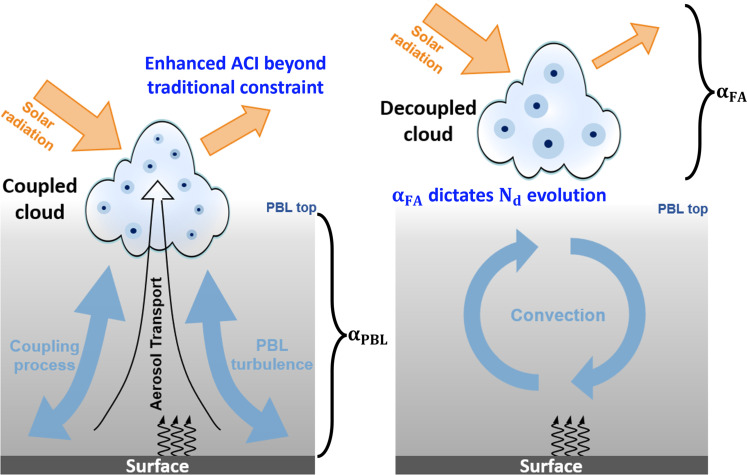
A schematic diagram describing the impacts of cloud coupling on ACIs. Orange arrows represent solar radiation. The gray shaded area indicates boundary layer aerosols. Black, curved arrows indicate surface heat fluxes. The coupling process, bridging clouds and the land surface, propels the vertical ascent of humidity, aerosols, and heat fluxes from the PBL up to the clouds (illustrated by the background black arrow). α_PBL_ and α_FA_, representing the aerosol loading within PBL and within free atmosphere, can be used as the aerosol proxies for coupled and decoupled regimes, respectively. Under the coupling regime, boundary layer aerosols cause a notable cooling effect through changing the cloud albedo. Under the decoupled regime, free atmosphere aerosols dictate the variations in cloud droplet number concentration (*N*_d_). As a net result, neglecting cloud coupling can result in an underestimation of aerosol indirect radiative forcing.

Our study highlights the important impact of cloud-surface coupling on ACI, a concept schematically presented in Fig. 5. Under a coupled regime, boundary layer aerosols, by facilitating the activation of more particles into cloud droplets, effectively increase *N*_d_, reduce the droplet effective radius, and hence entail notable effects of ACI. Conversely, in a decoupled regime, inefficient transport of aerosols from PBL to the cloud base leads to disconnection between cloud optical properties and boundary layer aerosols. It is primarily the aerosols from the free atmosphere that shape the *N*_d_ evolution under decoupled conditions.

Our discoveries shed light on the deeper intricacies of ACI processes and their radiative forcing implications. Traditional observations of RF_aci_ often blend the effects of coupled and decoupled clouds, leading to a potential bias in the quantification of ACI’s radiative impact. This is particularly evident when considering the weak responsiveness of *N*_d_ to standard aerosol proxies like AOD or surface aerosol loadings in decoupled conditions. Our research introduces a methodology that circumvents the direct retrieval of aerosol vertical profiles, addressing this important uncertainty in the RF_aci_ quantification. We observed that ACI in decoupled clouds is influenced primarily by free-atmosphere aerosols, resulting in markable cooling effects. To address this issue, we propose the use of aerosol metrics specific to the coupling state, which would offer a more accurate portrayal of cloud-surface interactions in ACI.

Observational and modeling estimates serve as the two pillars in assessing RF_aci_. Intergovernmental Panel on Climate Change (IPCC) assessments synthesize ACI estimations from both these two methods, as each comes with its own set of uncertainties and can have vast variations depending on the tools and datasets in use. Reconciling the discrepancies between model-based and observational estimates remains a complex and unresolved issue. Numerous studies indicate that climate models often produce higher effects of ACI compared to observational estimates, spanning from field observations (*50*, *51*) to satellite-derived regional means or global assessments (*14*, *16*, *21*, *52*). This study underscores cloud-surface coupling as a notable, yet not exclusive, factor contributing to these discrepancies. By considering cloud coupling, we present an approach for mitigating some of the biases associated with aerosol vertical structure prevalent in observational-based ACI estimates, thereby helping to bridge the existing gap between observational and model-based estimates. These findings have broad implications, as observation-derived sensitivity of cloud properties to aerosols usually serves as the reference for evaluating ACI effects in model simulations (*4*, *51*–*54*). Such findings suggest a need to improve the observational determination of the ACI, emphasizing the differentiation between coupled and decoupled cloud states. In addition to exploring cloud-surface coupling, this study acknowledges that discrepancies between model-based and observational RF_aci_ estimates arise from various factors, such as differences in dataset resolutions, aerosol compositions, model parameterizations, and model diversity (*15*).

It is also essential to note that observational and modeling tools are distinct entities, operating independently, which often precludes direct process-level comparisons between them. In climate models, averaged cloud properties span a 1° to 2° grid, characterized over daily or monthly time frames, to gauge ACI. Hence, outputs are not designed to discern the turbulent coupling between cloud and surface, the representation of cloud-surface coupling in climate models warrants detailed examination in the model parametrization.

Our study introduces an approach that underscores the critical role of cloud-surface coupling in ACI analysis, using specific regions as a foundational starting point. Accurate determination of cloud coupling states on a global scale remains an unexplored and substantial challenge. Notably, advancements in kilometer-scale climate models (*55*) and the advent of new satellite-based lidar systems (e.g., NASA Atmosphere Observing System) (*56*) present promising opportunities for global assessments of cloud coupling. Pursuing this direction has the potential to refine ACI quantification by tackling one of its major uncertainties.

## MATERIALS AND METHODS

### Descriptions of datasets

#### Beijing and SGP sites

This study uses extensive field observations obtained from a superstation in Beijing, China and observational data from the SGP observatory in Oklahoma. The measurements from the Beijing superstation encompass data gathered from ground meteorological instruments, PM_2.5_ data from the surrounding area, and micropulse lidar data, spanning the period of July 2017 to October 2019. Averaged PM_2.5_ concentrations were acquired from five air quality monitoring sites situated within a 20-km radius of the lidar site superstation. The SGP site in Oklahoma has been a rich source of precise observational data for climate research since the late 1980s. The datasets used in this study from the SGP site during October 1998 to December 2020 include (i) remote sensing products of cloud boundaries (*57*), (ii) vertical profiles of thermodynamic parameters, (iii) ground observing system of aerosols, (iv) cloud optical properties (i.e., cloud optical depth and effective radius) from the combined filed observations (multifilter rotating shadowband radiometer, microwave radiometer, and Langley analysis). Since measurements of cloud properties are only available in the SGP site, we use MODIS-derived cloud optical properties over the Eastern Asia to calculate the RF_aci_.

#### Aircraft data over the SGP

This study used in situ aerosol extinction from the IAP campaign (https://arm.gov/capabilities/instruments/iap), which includes an extensive collection of 627 flight missions spanning from 2000 to 2006 (*58*). We use the dry fine-mode aerosol extinction (diameter <1 μm) at the green channel (σ_dry_) from the aerosol observing system onboard the aircraft within a 15 km radius around the SGP site, focusing on measurements corresponding to 09:00 to 16:00 local times (LT) to align with the periods used for the RF_aci_ calculations.

To effectively represent the variations of aerosols in the subcloud layer, we normalize the measurement altitudes by the CBH and calculate the mean and SDs of aerosol extinction at intervals of 0.1 CBH. Smoothing across each level (0.1 CBH increment) within a ±1 level window was applied to separately average profiles below and above the cloud base. Both cloud positions and PBL height (PBLH) are still obtained from the ground-based instruments to match with IAP from aircraft. The mean aerosol extinction within the boundary layer (σ_PBL_) was computed using measurements below the PBL top within a ±1 hour window around each data point.

#### Radiosonde stations in eastern China

We also use radiosonde stations in eastern China to illuminate the influences of coupling between cloud and land surface on ACI. The China Meteorological Administration maintains the radiosonde sites, which measured vertical profiles of temperature, wind, moisture, and pressure, and wind at 14:00 LT during summer only. In this study, the characteristics of cloud-surface coupling were investigated using the 14:00 LT soundings from 2015 to 2019, excluding days with precipitation from 12:00 to 14:00 LT and sites with less than 100 valid soundings at 1400 LT. The location of 13 sites can be found in Fig. 4 and fig. S9.

#### MODIS cloud product

Because of the lack of ground measurements of cloud properties over Eastern Asia, we also use the MODIS Level-2 Cloud product, MYD06_L2 (5-Min L2 Swath 1 and 5 km). MODIS-derived cloud optical depth and cloud effective radius are used to investigate the responses of cloud properties to aerosols. We also use the MODIS cloud top height/temperature, cloud mask, cloud phase, liquid water path, and multilayer flag. Cloud properties are matched with ground observations within 20-km.

#### MERRA2 reanalysis data

Since the observational AOD is generally not available during cloudy conditions, we also used the AOD dataset obtained from MERRA2 at a spatial resolution of 0.5° × 0.625°. Furthermore, AOD_f_ is defined as the aggregate of AOD for black carbon, organic aerosols, and sulfate aerosols, along with 30% of sea salt aerosols. MEERA-2 also offers the 3-hour vertical distribution of aerosol mass mixing ratio of multiple species. The conversion from aerosol mass mixing ratio to PM and aerosol extinction (σ) is as follows

$$\text{PM}=\sum_{x=1}^{X}r_{x}\times \rho_{\text{air}}$$ (1)

$$\sigma =\sum_{x=1}^{X}\left(r_{x}\times \rho_{\text{air}}\right)\times m_{x}^{\text{ext}}$$ (2)

where *r_x_* is the aerosol mass mixing ratio of aerosol type *x*. ρ_air_ is the density of air and is provided by MERRA-2 for different atmospheric levels. $m_{x}^{\text{ext}}$ is the mass extinction coefficient for aerosol type *x*, indicating how much light is extinguished per unit mass of the aerosol. $m_{x}^{\text{ext}}$ for different aerosol species are documented in Randles *et al.* (*59*). We use the mass extinction coefficient at the dry condition to calculate σ_dry_. Following the MERRA-2 official website (https://gmao.gsfc.nasa.gov/reanalysis/MERRA-2/FAQ/), we excluded the mass mixing ratio from dust bins 2 to 5 and sea salt bins 3 to 5 for the calculation of fine-mode aerosol properties (diameter <1 μm), and included 70% of dust bin 1. Other species except dust and sea salt are considered as fine mode.

Table S1 compiles the necessary cloud, aerosol, and radiative parameters used for the data analyses and the computation of the RF_aci_. We also use the Max Planck Aerosol Climatology version 2 (MACv2) to assess aerosol anthropogenic fraction (*60*, *61*). The MACv2 defines monthly global maps for radiative properties and aerosol optical properties, with aerosol composition specifics derived via a top-down method that outlines the spectral aerosol single scattering properties. The determination of Anthropogenic AOD leverages scaling factors applied to the MACv2 fine-mode AOD values, which are based on model simulation results from AeroCom-Phase-2 using preindustrial (PI) and present-day (PD) emissions data.

### Cloud-surface coupling from lidar and radiosonde

This study used a method to illuminate coupling between clouds and the land surface using remote sensing techniques (*46*), specifically lidar and radiosonde measurements. The method is based on identifying the PBLH variability (*62*) and coupled states simultaneously by analyzing both the temporal continuity and vertical profiles of backscatter within the PBL obtained from lidar. Taking into account the temporal fluctuations of the PBL, the PBLH is determined as a step signal within the function of wavelet covariance transformation and signal gradient, derived from the backscatters of lidar. Using cloud boundary product, PBLH obtained from lidar, and lifted condensation level (*63*), we differentiate clouds into coupled or decoupled clouds. Clouds are classified as coupled when the turbulent flows originating from the ground level manage to reach the base of the cloud, thereby influencing its evolution, which results in a turbulence-facilitated linkage among surface fluxes, PBL, and the cloud. For coupled clouds, we can estimate the PBLH based on cloud location. The cloud top height can be regarded as the PBL top for stratiform clouds pending on conditions, and for active cumulus clouds, the CBH is used to derive the PBLH.

In addition, we assimilate radiosonde data to provide the standard identification of coupling between clouds and the land surface whenever the measurements are available. On the basis of the potential temperature profiles obtained from radiosonde data, we determine cloud-surface coupling. When a potential temperature inversion exists between the cloud base and the PBL top, we classify the cloud as decoupled; if no such inversion exists, we consider the cloud as coupled with the surface. Specifically, we adopted the Liu and Liang method (*64*) to derive PBL top from radiosonde data. We implement a cloud layer identification methodology, devised by Zhang *et al.* (*65*), that uses three distinct height-resolving relative humidity thresholds to identify the various cloud stratifications. In general, the lidar-based method shows reasonably well consistency with radiosonde-derived cloud-surface coupling with about 10% omission errors and commission errors (*46*).

### Calculation of aerosol vertical distributions from lidar

Aerosol vertical distributions were calculated from the micropulse lidar over Beijing using the Klett method (*66*) to retrieve vertical profiles of aerosol extinction from the lidar signals at 532 nm. The column-averaged lidar ratio, a critical parameter for retrieving extinction profiles, was normalized using AOD at 0.5 μm derived from Aerosol Robotic Network. For cloudy conditions, linear interpolation of the lidar ratio was applied. At the SGP site, we used aerosol extinction profiles from Raman lidar operating at 355 nm, which provides additional constraints for retrieving aerosol extinction profiles.

To account for the substantial overlap effect near the surface (*67*), we use the aerosol extinction profiles derived from lidar above 0.3 km. The aerosol extinction coefficient was assumed to be constant within the blind zone. Accounting for multiple scattering effects, an overall uncertainty of 30% was observed during the retrieval of aerosol extinction (*68*). To estimate aerosol loading below the cloud base, we calculated the average aerosol extinctions derived from adjacent clear pixels at the CBH within 2 hours. To avoid cloud contamination, clear pixels are defined as those distanced more than 0.3 km from the cloud base. AOD_sub_ is determined by multiplying the mean aerosol extinction in the subcloud layer by either the CBH or 0.3 km if the cloud base resides below this threshold. Clouds introduced additional noise in the aerosol profiles; thus, we used adjacent clear-sky aerosol profiles within a 2-hour window as a proxy to analyze AOD_sub_. If no adjacent clear-sky aerosol profiles were available, we used cloudy aerosol profiles instead.

### Methodology for calculating RF_aci_

#### Observational approach

RF_aci_ is calculated based on the change in *N*_d_ since the industrial revolution (∆*N*_d_). We calculate the relative change of *N*_d_ from the PI era to PD using a coefficient relating lnα to ln*N*_d_ as follows

$$\frac{∆N_{d}}{N_{d}}=[1-\frac{{\left(N_{d}\right)}_{\text{PI}}}{{\left(N_{d}\right)}_{\text{PD}}}]=\left[1-{\left(\frac{\alpha_{\text{PI}}}{\alpha_{\text{PD}}}\right)}^{\beta }\right]$$ (3)

where β is the $\frac{d\text{ln}N_{d}}{d\text{ln}\alpha }$ and is calculated as a linear regression between *N*_d_ and aerosol proxy, α. The confidence level for the linear regression was calculated using the coefficient confidence intervals from the linear model fit (*69*). α_PI_ and α_PD_ indicate the aerosol loading for the PI and PD, respectively. In this study, we use AOD_f_ or fine-mode aerosol extinction (σ) as the aerosol proxy to calculate the RF_aci_. These aerosol proxies, intended to represent CCN at the cloud base, carry notable uncertainties that contribute to the overall uncertainty in estimating RF_aci_ (*37*, *70*). The ratio between α_PD_ and α_PI_ is calculated as the ratio in AOD_f_ between PD and PI derived from MACv2 (fig. S10).

Charlson *et al.* (*71*) proposed a method to estimate of the RF_aci_ for liquid clouds, which has been used in numerous studies (*18*, *27*). They suggested that RF_aci_ (∆*F*^↑^) for anthropogenic aerosols can be expressed as a function of liquid water cloud fraction, *f*_liq_, the cloud albedo, *A*_liq_, cloud droplet number concentration, *N*_d_, aerosol proxy, α, and daily mean down-welling solar flux, *F*^↓^.

$$∆F^{↑}=-\frac{1}{3}F^{↓}f_{\text{liq}}A_{\text{liq}}\left(1-A_{\text{liq}}\right)\frac{∆N_{d}}{N_{d}}$$ (4)

where *F*^↓^ is obtained from the European Centre for Medium-Range Weather Forecasts Reanalysis v5 (ERA-5) data (*72*). Following Segrin *et al.* (*73*), we calculate A_liq_ as follows

$$A_{\text{liq}}=\frac{\frac{3}{4}\left(1-g\right)\tau }{1+\frac{3}{4}\left(1-g\right)\tau }$$ (5)

where τ is cloud optical depth and the asymmetry parameter *g* is assumed to be 0.85 (*49*).

The values of RF_aci_ for coupled and decoupled regimes also are analyzed separately in our study.

$$\text{Coupled regime}:{\left(∆F^{↑}\right)}_{\text{coupled}}=-\frac{1}{3}f_{\text{co}}F^{↓}f_{\text{liq}}A_{\text{liq}}\left(1-A_{\text{liq}}\right)\frac{∆N_{d}}{N_{d}}$$ (6)

$$\text{Decoupled regime}:{\left(∆F^{↑}\right)}_{\text{decoupled}}=-\frac{1}{3}f_{\text{de}}F^{↓}f_{\text{liq}}A_{\text{liq}}\left(1-A_{\text{liq}}\right)\frac{∆N_{d}}{N_{d}}$$ (7)

where *f*_co_ denotes the coupled fraction, which indicates the proportion of coupled clouds within liquid water clouds. *f*_de_ represents the decoupled fraction (*f*_de_ = 1 − *f*_co_). The climatology of cloud-surface coupling and coupling fraction are represented in figs. S4 and S11, respectively. *A*_liq_ is separately averaged for coupled and decoupled regimes. The mixture of coupled and decoupled clouds within 1 hour is removed. The confidence level for RF_aci_ is derived from the confidence interval of linear regression β. RF_aci_ can be considered as the sum of (∆*F*^↑^)_coupled_ and (∆*F*^↑^)_decoupled_.

To calculate RF_aci_, an important step is the assessment of the variation in *N*_d_ as a result of anthropogenic aerosols, leveraging the relationship between *N*_d_ and α, denoted as $\frac{d\text{ln}N_{d}}{d\text{ln}\alpha }$. *N*_d_ is derived from the cloud effective radius and cloud optical depth for liquid water clouds, under the assumption of an adiabatic condition.

$$N_{d}=\gamma \tau_{c}^{1/2}r_{e}^{-5/2}$$ (8)

The cloud properties are derived from field observations during 09:00 to 16:00 LT over the SGP (*74*) and are derived from Aqua MODIS over the Eastern Asia. $\frac{d\text{ln}N_{d}}{d\text{ln}\alpha }$ was calculated for different regions. For the Eastern Asia region, we use the cloud phase product from MODIS to identify and select only liquid water clouds. For the SGP region, clouds are analyzed in our analysis only when the hourly cloud top temperature exceeds 273 K. The temperature profiles used to determine this criterion are sourced from ERA-5. For more reliable fitting parameters, statistical regressions are performed using a subset of data that exhibits lower retrieval inaccuracies. Following the previous studies (*16*, *27*, *75*), our analyses exclude the retrievals involving multilayered clouds (MODIS product), thin clouds (liquid water path, *L* < 20 g m^−2^), and possible precipitable clouds (*L* > 200 g m^−2^). Furthermore, the bottom 15% of data for aerosol loading (α) are also excluded due to the sensitivity of the slopes of In *N*_d_ versus In α to minimal aerosol variations. These minor changes have notably large retrieval uncertainties for both AOD and aerosol extinction (*16*, *76*). As for the calculation of RF_aci_, all liquid water clouds are used to calculate the liquid water cloud fraction and the cloud optical depth (*16*, *25*). All datasets are averaged with an hourly resolution. Because of the data availability, we rely on cloud properties obtained from MODIS measurements over the Eastern Asian during the daytime.

#### Modeling output

This study also directly used the output of RF_aci_ from the work of Yu *et al.* (*19*), which integrates a size-resolved advanced particle microphysics model and the rapid radiative transfer model for GCMs for shortwave radiation with the GEOS-Chem model. This modeling approach explicitly simulates the formation, growth, and atmospheric processing of secondary and primary aerosols, including sulfate, nitrate, ammonium, secondary organic aerosol, black carbon, primary organic carbon, dust, and sea salt particles. This modeling approach assesses aerosol impacts on cloud albedo and solar radiation by comparing simulations with PD emissions against preindustrial conditions. The approach for estimating RF_aci_ follows IPCC guidelines, isolating the effect of increased aerosol concentration on cloud optical properties and top-of-atmosphere solar fluxes, without considering feedback mechanisms.

## References

[1] O. Boucher, D. Randall, P. Artaxo, C. Bretherton, G. Feingold, P. Forster, V.-M. Kerminen, Y. Kondo, H. Liao, U. Lohmann, "Clouds and Aerosols" in *Climate Change 2013: The Physical Science Basis*. *Contribution of Working Group I to the Fifth Assessment Report of the Intergovernmental Panel on Climate Change* (Cambridge Univ. Press, 2014), pp. 571–657.

[2] S. Solomon, D. Qin, M. Manning, K. Averyt, M. Marquis, *Climate change 2007-the physical science basis: Working group I contribution to the fourth assessment report of the IPCC* (Cambridge Univ. Press, 2007), vol. 4.

[3] V. Masson-Delmotte, P. Zhai, A. Pirani, S. L. Connors, C. Péan, S. Berger, N. Caud, Y. Chen, L. Goldfarb, M. Gomis, Climate change 2021: The physical science basis. Contribution of working group I to the sixth assessment report of the intergovernmental panel on climate change 2, (2021).

[4] F. F. Malavelle, J. M. Haywood, A. Jones, A. Gettelman, L. Clarisse, S. Bauduin, R. P. Allan, I. H. H. Karset, J. E. Kristjánsson, L. Oreopoulos, Strong constraints on aerosol–cloud interactions from volcanic eruptions. Nature 546, 485–491 (2017). 28640263 10.1038/nature22974

[5] Y.-T. Hwang, S.-P. Xie, P.-J. Chen, H.-Y. Tseng, C. Deser, Contribution of anthropogenic aerosols to persistent La Niña-like conditions in the early 21st century. Proc. Natl. Acad. Sci. U.S.A. 121, e2315124121 (2024). 38252827 10.1073/pnas.2315124121 PMC10835045

[6] S. Twomey, Pollution and the planetary albedo. Atmos. Environ. 8, 1251–1256 (1974).

[7] B. A. Albrecht, Aerosols, cloud microphysics, and fractional cloudiness. Science 245, 1227–1230 (1989). 17747885 10.1126/science.245.4923.1227

[8] Z. Li, W. M. Lau, V. Ramanathan, G. Wu, Y. Ding, M. Manoj, J. Liu, Y. Qian, J. Li, T. Zhou, Aerosol and monsoon climate interactions over Asia. Rev. Geophys. 54, 866–929 (2016).

[9] Z. Li, F. Niu, J. Fan, Y. Liu, D. Rosenfeld, Y. Ding, Long-term impacts of aerosols on the vertical development of clouds and precipitation. Nat. Geosci. 4, 888–894 (2011).

[10] R. Wood, M. Wyant, C. S. Bretherton, J. Rémillard, P. Kollias, J. Fletcher, J. Stemmler, S. De Szoeke, S. Yuter, M. Miller, Clouds, aerosols, and precipitation in the marine boundary layer: An arm mobile facility deployment. B. Am. Meteorol. Soc. 96, 419–440 (2015).

[11] S.-P. Xie, B. Lu, B. Xiang, Similar spatial patterns of climate responses to aerosol and greenhouse gas changes. Nat. Geosci. 6, 828–832 (2013).

[12] T. Yuan, H. Song, R. Wood, L. Oreopoulos, S. Platnick, C. Wang, H. Yu, K. Meyer, E. Wilcox, Observational evidence of strong forcing from aerosol effect on low cloud coverage. Sci. Adv. 9, eadh7716 (2023). 37939179 10.1126/sciadv.adh7716 PMC12488053

[13] J. R. French, K. Friedrich, S. A. Tessendorf, R. M. Rauber, B. Geerts, R. M. Rasmussen, L. Xue, M. L. Kunkel, D. R. Blestrud, Precipitation formation from orographic cloud seeding. Proc. Natl. Acad. Sci. U.S.A. 115, 1168–1173 (2018). 29358387 10.1073/pnas.1716995115 PMC5819430

[14] U. Lohmann, G. Lesins, Stronger constraints on the anthropogenic indirect aerosol effect. Science 298, 1012–1015 (2002). 12411701 10.1126/science.1075405

[15] N. Bellouin, J. Quaas, E. Gryspeerdt, S. Kinne, P. Stier, D. Watson-Parris, O. Boucher, K. S. Carslaw, M. Christensen, A. L. Daniau, J. L. Dufresne, G. Feingold, S. Fiedler, P. Forster, A. Gettelman, J. M. Haywood, U. Lohmann, F. Malavelle, T. Mauritsen, D. T. McCoy, G. Myhre, J. Mulmenstadt, D. Neubauer, A. Possner, M. Rugenstein, Y. Sato, M. Schulz, S. E. Schwartz, O. Sourdeval, T. Storelvmo, V. Toll, D. Winker, B. Stevens, Bounding global aerosol radiative forcing of climate change. Rev. Geophys. 58, e2019RG000660 (2020). 10.1029/2019RG000660 PMC7384191 32734279

[16] H. Jia, X. Ma, F. Yu, J. Quaas, Significant underestimation of radiative forcing by aerosol–cloud interactions derived from satellite-based methods. Nat. Commun. 12, 3649 (2021). 34131118 10.1038/s41467-021-23888-1 PMC8206093

[17] J. Quaas, O. Boucher, N. Bellouin, S. Kinne, Satellite-based estimate of the direct and indirect aerosol climate forcing. J. Geophys. Res. Atmos. 113, 8962 (2008).

[18] D. McCoy, F. M. Bender, J. Mohrmann, D. Hartmann, R. Wood, D. Grosvenor, The global aerosol-cloud first indirect effect estimated using MODIS, MERRA, and AeroCom. J. Geophys. Res. Atmos. 122, 1779–1796 (2017).

[19] F. Yu, X. Ma, G. Luo, Anthropogenic contribution to cloud condensation nuclei and the first aerosol indirect climate effect. Environ. Res. Lett. 8, 024029 (2013).

[20] Y.-C. Chen, M. W. Christensen, G. L. Stephens, J. H. Seinfeld, Satellite-based estimate of global aerosol–cloud radiative forcing by marine warm clouds. Nat. Geosci. 7, 643–646 (2014).

[21] J. E. Penner, L. Xu, M. Wang, Satellite methods underestimate indirect climate forcing by aerosols. Proc. Natl. Acad. Sci. U.S.A. 108, 13404–13408 (2011). 21808047 10.1073/pnas.1018526108 PMC3158199

[22] D. Rosenfeld, Y. Zheng, E. Hashimshoni, M. L. Pöhlker, A. Jefferson, C. Pöhlker, X. Yu, Y. Zhu, G. Liu, Z. Yue, Satellite retrieval of cloud condensation nuclei concentrations by using clouds as CCN chambers. Proc. Natl. Acad. Sci. U.S.A. 113, 5828–5834 (2016). 26944081 10.1073/pnas.1514044113 PMC4889349

[23] D. Rosenfeld, Aerosol-driven droplet concentrations dominate coverage and water of oceanic low-level clouds (vol 364, eaay4194, 2019). Science 365, 230–230 (2019). 30655446 10.1126/science.aav0566

[24] J. Fan, Y. Wang, D. Rosenfeld, X. Liu, Review of aerosol–cloud interactions: Mechanisms, significance, and challenges. J. Atmos. Sci. 73, 4221–4252 (2016).

[25] J. Quaas, Approaches to observe anthropogenic aerosol-cloud interactions. Curr. Clim. Change Rep. 1, 297–304 (2015). 26618102 10.1007/s40641-015-0028-0 PMC4654431

[26] Y. Wang, X. Zheng, X. Dong, B. Xi, P. Wu, T. Logan, Y. L. Yung, Impacts of long-range transport of aerosols on marine-boundary-layer clouds in the eastern North Atlantic. Atmos. Chem. Phys. 20, 14741–14755 (2020).

[27] E. Gryspeerdt, J. Quaas, S. Ferrachat, A. Gettelman, S. Ghan, U. Lohmann, H. Morrison, D. Neubauer, D. G. Partridge, P. Stier, Constraining the instantaneous aerosol influence on cloud albedo. Proc. Natl. Acad. Sci. U.S.A. 114, 4899–4904 (2017). 28446614 10.1073/pnas.1617765114 PMC5441736

[28] M. W. Christensen, A. Gettelman, J. Cermak, G. Dagan, M. Diamond, A. Douglas, G. Feingold, F. Glassmeier, T. Goren, D. P. Grosvenor, Opportunistic experiments to constrain aerosol effective radiative forcing. Atmos. Chem. Phys. 22, 641–674 (2022). 35136405 10.5194/acp-22-641-2022 PMC8819675

[29] X. Zheng, B. Xi, X. Dong, T. Logan, Y. Wang, P. Wu, Investigation of aerosol–cloud interactions under different absorptive aerosol regimes using Atmospheric Radiation Measurement (ARM) southern Great Plains (SGP) ground-based measurements. Atmos. Chem. Phys. 20, 3483–3501 (2020).

[30] J. Liu, Z. Li, Estimation of cloud condensation nuclei concentration from aerosol optical quantities: Influential factors and uncertainties. Atmos. Chem. Phys. 14, 471–483 (2014).

[31] V. Toll, M. Christensen, J. Quaas, N. Bellouin, Weak average liquid-cloud-water response to anthropogenic aerosols. Nature 572, 51–55 (2019). 31367029 10.1038/s41586-019-1423-9

[32] D. Painemal, F.-L. Chang, R. Ferrare, S. Burton, Z. Li, W. L. Smith Jr., P. Minnis, Y. Feng, M. Clayton, Reducing uncertainties in satellite estimates of aerosol–cloud interactions over the subtropical ocean by integrating vertically resolved aerosol observations. Atmos. Chem. Phys. 20, 7167–7177 (2020).

[33] N. Wang, K. Zhang, X. Shen, Y. Wang, J. Li, C. Li, J. Mao, A. Malinka, C. Zhao, L. M. Russell, Dual-field-of-view high-spectral-resolution lidar: Simultaneous profiling of aerosol and water cloud to study aerosol–cloud interaction. Proc. Natl. Acad. Sci. U.S.A. 119, e2110756119 (2022). 35235447 10.1073/pnas.2110756119 PMC8915832

[34] S. J. Ghan, T. A. Rissman, R. Elleman, R. A. Ferrare, D. Turner, C. Flynn, J. Wang, J. Ogren, J. Hudson, H. H. Jonsson, Use of in situ cloud condensation nuclei, extinction, and aerosol size distribution measurements to test a method for retrieving cloud condensation nuclei profiles from surface measurements. J. Geophys. Res. Atmos. 111, 5752 (2006).

[35] M. Lv, Z. Wang, Z. Li, T. Luo, R. Ferrare, D. Liu, D. Wu, J. Mao, B. Wan, F. Zhang, Retrieval of cloud condensation nuclei number concentration profiles from lidar extinction and backscatter data. J. Geophys. Res. Atmos. 123, 6082–6098 (2018).

[36] R.-E. Mamouri, A. Ansmann, Potential of polarization lidar to provide profiles of CCN- and INP-relevant aerosol parameters. Atmos. Chem. Phys. 16, 5905–5931 (2016).

[37] J. Quaas, A. Arola, B. Cairns, M. Christensen, H. Deneke, A. M. Ekman, G. Feingold, A. Fridlind, E. Gryspeerdt, O. Hasekamp, Constraining the Twomey effect from satellite observations: Issues and perspectives. Atmos. Chem. Phys. 20, 15079–15099 (2020).

[38] L. Costantino, F. M. Bréon, Analysis of aerosol-cloud interaction from multi-sensor satellite observations. Geophys. Res. Lett. 37, (2010).

[39] L. Costantino, F.-M. Bréon, Aerosol indirect effect on warm clouds over South-East Atlantic, from co-located MODIS and CALIPSO observations. Atmos. Chem. Phys. 13, 69–88 (2013).

[40] D. Painemal, J. Y. C. Chiu, P. Minnis, C. Yost, X. Zhou, M. Cadeddu, E. Eloranta, E. R. Lewis, R. Ferrare, P. Kollias, Aerosol and cloud microphysics covariability in the northeast Pacific boundary layer estimated with ship-based and satellite remote sensing observations. J. Geophys. Res. Atmos. 122, 2403–2418 (2017).

[41] T. Su, Z. Li, Y. Zheng, Cloud-surface coupling alters the morning transition from stable to unstable boundary layer. Geophys. Res. Lett. 50, e2022GL102256 (2023).

[42] C. Jones, C. Bretherton, D. Leon, Coupled vs. decoupled boundary layers in VOCALS-REx. Atmos. Chem. Phys. 11, 7143–7153 (2011).

[43] S. Nicholls, The dynamics of stratocumulus: Aircraft observations and comparisons with a mixed layer model. Q. J. Roy. Meteor. Soc. 110, 783–820 (1984).

[44] H. J. Griesche, K. Ohneiser, P. Seifert, M. Radenz, R. Engelmann, A. Ansmann, Contrasting ice formation in Arctic clouds: Surface-coupled vs. surface-decoupled clouds. Atmos. Chem. Phys. 21, 10357–10374 (2021).

[45] L. Delle Monache, K. D. Perry, R. T. Cederwall, J. A. Ogren, In situ aerosol profiles over the Southern Great Plains cloud and radiation test bed site: 2. Effects of mixing height on aerosol properties. J. Geophys. Res. Atmos. 109, 4024 (2004).

[46] T. Su, Y. Zheng, Z. Li, Methodology to determine the coupling of continental clouds with surface and boundary layer height under cloudy conditions from lidar and meteorological data. Atmos. Chem. Phys. 22, 1453–1466 (2022).

[47] R. Gelaro, W. McCarty, M. J. Suárez, R. Todling, A. Molod, L. Takacs, C. A. Randles, A. Darmenov, M. G. Bosilovich, R. Reichle, The modern-era retrospective analysis for research and applications, version 2 (MERRA-2). J. Climate 30, 5419–5454 (2017). 10.1175/JCLI-D-16-0758.1 PMC6999672 32020988

[48] X. Ma, F. Yu, J. Quaas, Reassessment of satellite-based estimate of aerosol climate forcing. J. Geophys. Res. Atmos. 119, 10,394-310,409 (2014).

[49] M. S. Diamond, H. M. Director, R. Eastman, A. Possner, R. Wood, Substantial cloud brightening from shipping in subtropical low clouds. AGU Adv. 1, e2019AV000111 (2020).

[50] A. C. Varble, P.-L. Ma, M. W. Christensen, J. Mülmenstädt, S. Tang, J. Fast, Evaluation of liquid cloud albedo susceptibility in E3SM using coupled eastern North Atlantic surface and satellite retrievals. Atmos. Chem. Phys. 23, 13523–13553 (2023).

[51] S. Tang, A. C. Varble, J. D. Fast, K. Zhang, P. Wu, X. Dong, F. Mei, M. Pekour, J. C. Hardin, P.-L. Ma, Earth System Model Aerosol–Cloud Diagnostics (ESMAC Diags) package, version 2: Assessing aerosols, clouds, and aerosol–cloud interactions via field campaign and long-term observations. Geosci. Model Dev. 16, 6355–6376 (2023).

[52] G. Saponaro, M. K. Sporre, D. Neubauer, H. Kokkola, P. Kolmonen, L. Sogacheva, A. Arola, G. De Leeuw, I. H. Karset, A. Laaksonen, Evaluation of aerosol and cloud properties in three climate models using MODIS observations and its corresponding COSP simulator, as well as their application in aerosol–cloud interactions. Atmos. Chem. Phys. 20, 1607–1626 (2020).

[53] D. Rosenfeld, M. O. Andreae, A. Asmi, M. Chin, G. de Leeuw, D. P. Donovan, R. Kahn, S. Kinne, N. Kivekäs, M. Kulmala, Global observations of aerosol-cloud-precipitation-climate interactions. Rev. Geophys. 52, 750–808 (2014).

[54] A. Bodas-Salcedo, J. Mulcahy, T. Andrews, K. Williams, M. Ringer, P. Field, G. Elsaesser, Strong dependence of atmospheric feedbacks on mixed-phase microphysics and aerosol-cloud interactions in HadGEM3. J. Adv. Model. Earth Syst. 11, 1735–1758 (2019). 31598189 10.1029/2019MS001688 PMC6774284

[55] P. M. Caldwell, C. R. Terai, B. Hillman, N. D. Keen, P. Bogenschutz, W. Lin, H. Beydoun, M. Taylor, L. Bertagna, A. Bradley, Convection-permitting simulations with the E3SM global atmosphere model. J. Adv. Model. Earth Syst. 13, e2021MS002544 (2021).

[56] S. A. Braun, J. Yorks, T. Thorsen, D. Cecil, D. Kirschbaum, in *IGARSS 2022–2022 IEEE International Geoscience and Remote Sensing Symposium*. (IEEE, 2022), pp. 7391–7393.

[57] E. E. Clothiaux, T. P. Ackerman, G. G. Mace, K. P. Moran, R. T. Marchand, M. A. Miller, B. E. Martner, Objective determination of cloud heights and radar reflectivities using a combination of active remote sensors at the ARM CART sites. J. Appl. Meteorol. Climatol. 39, 645–665 (2000).

[58] E. Andrews, P. Sheridan, J. Ogren, Seasonal differences in the vertical profiles of aerosol optical properties over rural Oklahoma. Atmos. Chem. Phys. 11, 10661–10676 (2011).

[59] C. A. Randles, A. M. Da Silva, V. Buchard, P. R. Colarco, A. Darmenov, R. Govindaraju, A. Smirnov, B. Holben, R. Ferrare, J. Hair, Y. Shinozuka, C. J. Flynn, The MERRA-2 aerosol reanalysis, 1980 onward. Part I: System description and data assimilation evaluation. J. Climate 30, 6823–6850 (2017). 10.1175/JCLI-D-16-0609.1 PMC5859955 29576684

[60] S. Kinne, The MACv2 aerosol climatology. Tellus B: Chem.Phys. Meteorol. 71, 1623639–1623621 (2022).

[61] B. Stevens, S. Fiedler, S. Kinne, K. Peters, S. Rast, J. Müsse, S. J. Smith, T. Mauritsen, MACv2-SP: A parameterization of anthropogenic aerosol optical properties and an associated Twomey effect for use in CMIP6. Geosci. Model Dev. 10, 433–452 (2017).

[62] T. Su, Z. Li, R. Kahn, A new method to retrieve the diurnal variability of planetary boundary layer height from lidar under different thermodynamic stability conditions. Remote Sens. Environ. 237, 111519 (2020).

[63] D. M. Romps, Exact expression for the lifting condensation level. J. Atmos. Sci. 74, 3891–3900 (2017).

[64] S. Liu, X.-Z. Liang, Observed diurnal cycle climatology of planetary boundary layer height. J. Climate 23, 5790–5809 (2010).

[65] J. Zhang, Z. Li, H. Chen, M. Cribb, Validation of a radiosonde-based cloud layer detection method against a ground-based remote sensing method at multiple ARM sites. J. Geophys. Res. Atmos. 118, 846–858 (2013).

[66] J. D. Klett, Lidar inversion with variable backscatter extinction ratios. Appl. Optics 24, 1638–1643 (1985). 10.1364/ao.24.001638 18223768

[67] A. Ansmann, M. Riebesell, U. Wandinger, C. Weitkamp, E. Voss, W. Lahmann, W. Michaelis, Combined Raman elastic-backscatter lidar for vertical profiling of moisture, aerosol extinction, backscatter, and lidar ratio. Appl. Phys. B 55, 18–28 (1992).

[68] Q. S. He, C. C. Li, J. T. Mao, A. K. H. Lau, P. R. Li, A study on the aerosol extinction-to-backscatter ratio with combination of micro-pulse LIDAR and MODIS over Hong Kong. Atmos. Chem. Phys. 6, 3243–3256 (2006).

[69] J. Sun, C. R. Loader, Simultaneous confidence bands for linear regression and smoothing. Annal. Stat. 22, 1328–1345 (1994).

[70] P. Stier, Limitations of passive remote sensing to constrain global cloud condensation nuclei. Atmos. Chem. Phys. 16, 6595–6607 (2016).

[71] R. J. Charlson, S. Schwartz, J. Hales, R. D. Cess, J. Coakley Jr., J. Hansen, D. Hofmann, Climate forcing by anthropogenic aerosols. Science 255, 423–430 (1992). 17842894 10.1126/science.255.5043.423

[72] H. Hersbach, B. Bell, P. Berrisford, S. Hirahara, A. Horányi, J. Muñoz-Sabater, J. Nicolas, C. Peubey, R. Radu, D. Schepers, The ERA5 global reanalysis. Q. J. Roy. Meteor. Soc. 146, 1999–2049 (2020).

[73] M. S. Segrin, J. A. Coakley Jr., W. R. Tahnk, MODIS observations of ship tracks in summertime stratus off the west coast of the United States. J. Atmos. Sci. 64, 4330–4345 (2007).

[74] D. D. Turner, S. McFarlane, L. Riihimaki, Y. Shi, C. Lo, Q. Min, "Cloud Optical Properties from the Multifilter Shadowband Radiometer (MFRSRCLDOD). An ARM Value-Added Product" (DOE ARM Climate Research Facility, Washington, DC (United States), 2014).

[75] O. P. Hasekamp, E. Gryspeerdt, J. Quaas, Analysis of polarimetric satellite measurements suggests stronger cooling due to aerosol-cloud interactions. Nat. Commun. 10, 5405 (2019). 31776336 10.1038/s41467-019-13372-2 PMC6881401

[76] A. Arola, A. Lipponen, P. Kolmonen, T. H. Virtanen, N. Bellouin, D. P. Grosvenor, E. Gryspeerdt, J. Quaas, H. Kokkola, Aerosol effects on clouds are concealed by natural cloud heterogeneity and satellite retrieval errors. Nat. Commun. 13, 7357 (2022). 36446763 10.1038/s41467-022-34948-5 PMC9708656

