# Supplementary material for: Constraining effects of aerosol-cloud interaction by accounting for coupling between cloud and land surface
Source: Sci Adv. 2024 May 23;10(21):eadl5044. doi: 10.1126/sciadv.adl5044 (PMC11114194; doi:10.1126/sciadv.adl5044)
Supplement: Supplementary file 1 — Figs. S1 to S11 Table S1 [file sciadv.adl5044_sm.pdf]

Science Advances
AAAS

Supplementary Materials for

Constraining effects of aerosol-cloud interaction by accounting for coupling between cloud and land surface

Tianning Su *et al.*

Corresponding author: Zhanqing Li, zhanqing@umd.edu; Tianning Su, su10@llnl.gov

Sci. Adv. **10**, eadl5044 (2024)
DOI: 10.1126/sciadv.adl5044

This PDF file includes:

Figs. S1 to S11
Table S1

Supplementary Materials

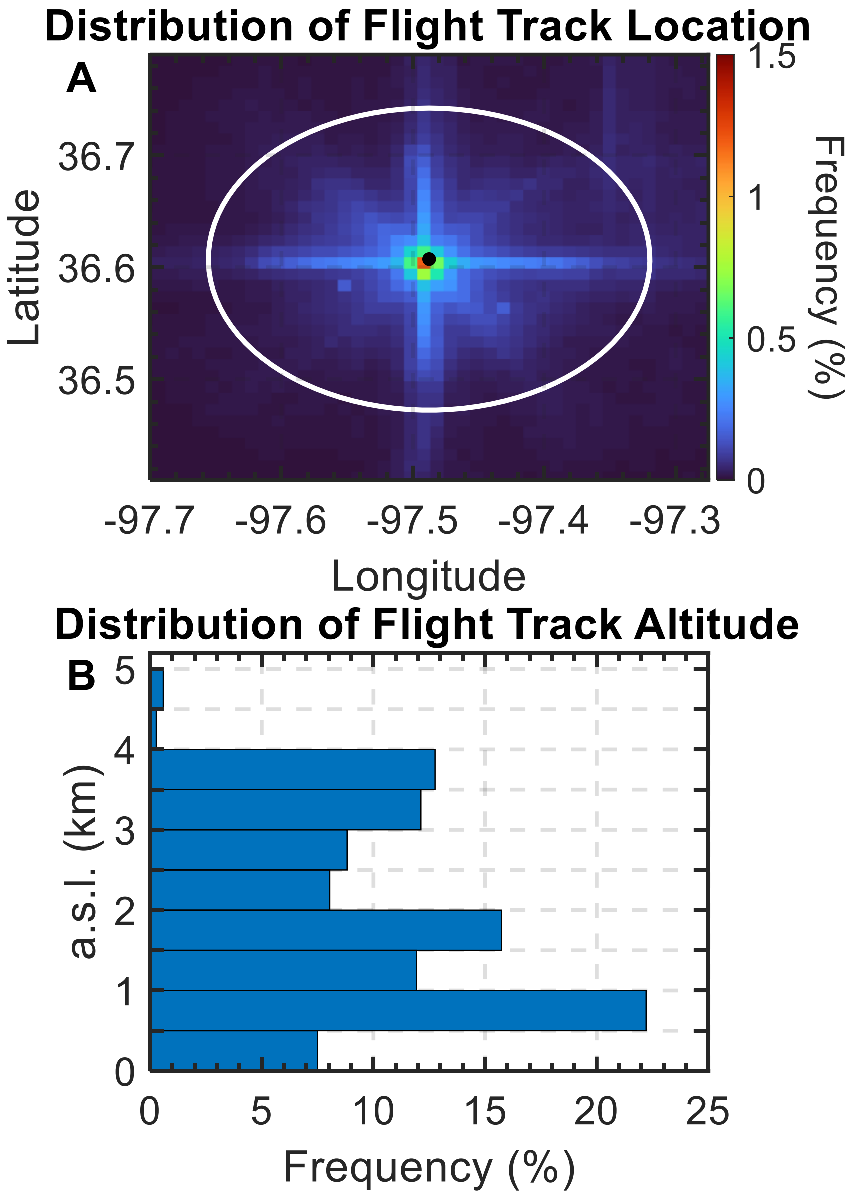

Fig. S1. Spatial and vertical sampling frequency of aerosol measurements from aircraft missions. (A) Heatmap of aircraft sampling frequency over the Southern Great Plains, showing the geographical frequency of flight paths during 627 missions during The In-Situ Aerosol Profiles (IAP) campaign from 2000 to 2006 (https://adc.arm.gov/discovery/#/results/instrument_class_code::iap). The white circle indicates the 15-km distance around the central site (black dot). (B) Vertical profile of aerosol sampling frequency, illustrating the distribution of aerosol measurements across different altitudes above the sea level (a.s.l.) up to 4 km. The bar lengths represent the percentage of samples taken at corresponding altitude during the flight missions.

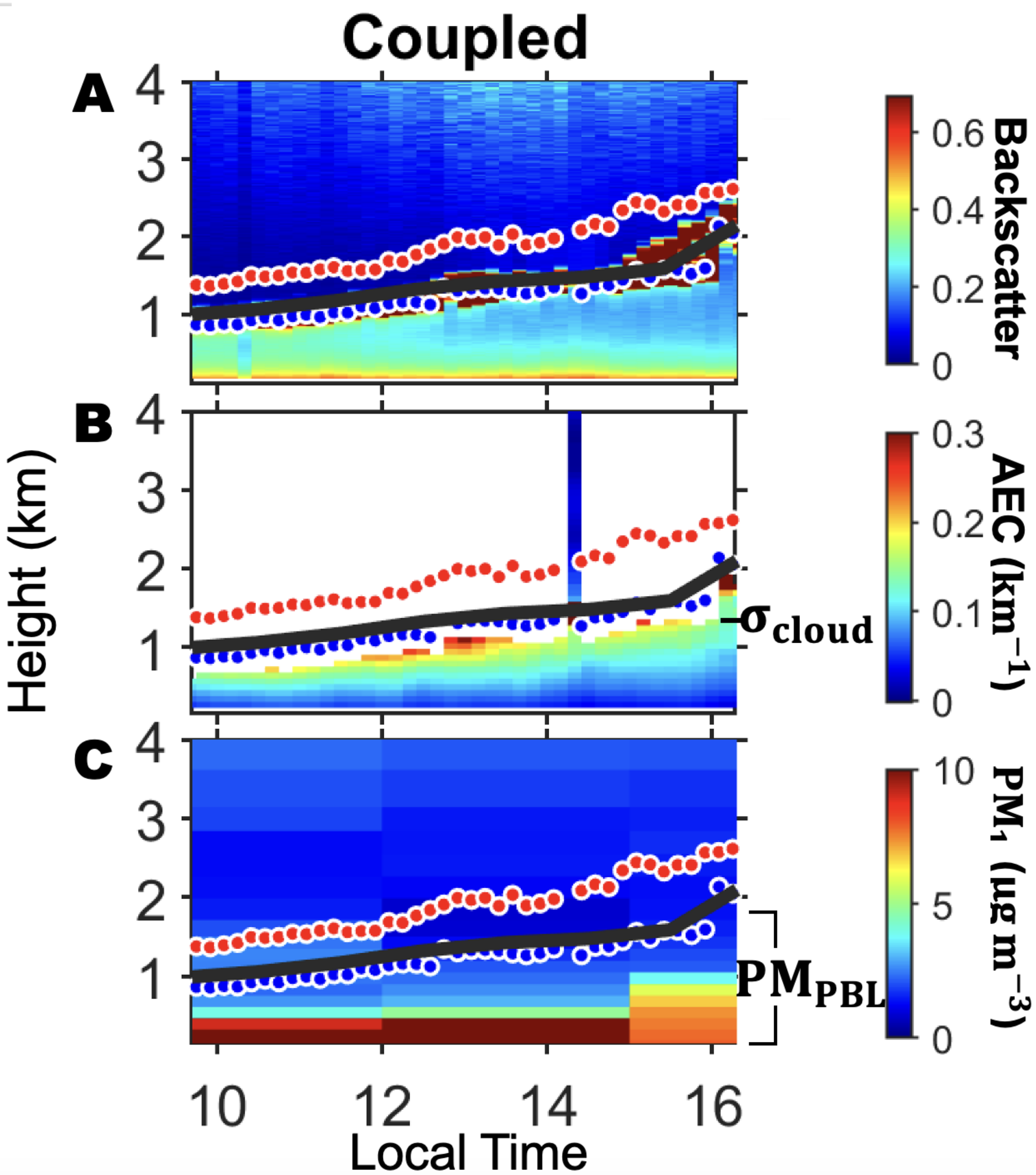

Fig. S2. A coupled cloud case. (A) Lidar backscatter profiles, (B) Aerosol Extinction Coefficients (AEC) profiles derived from Raman Lidar, and (C) PM_1 profiles obtained from MERRA-2. The cloud base and cloud top are represented by blue and red dots, respectively. The position of the PBL top is marked by a black line. σ_{cloud} indicates the aerosol extinction below the cloud base, while PM_{PBL} denotes the mean PM_1 within the PBL.

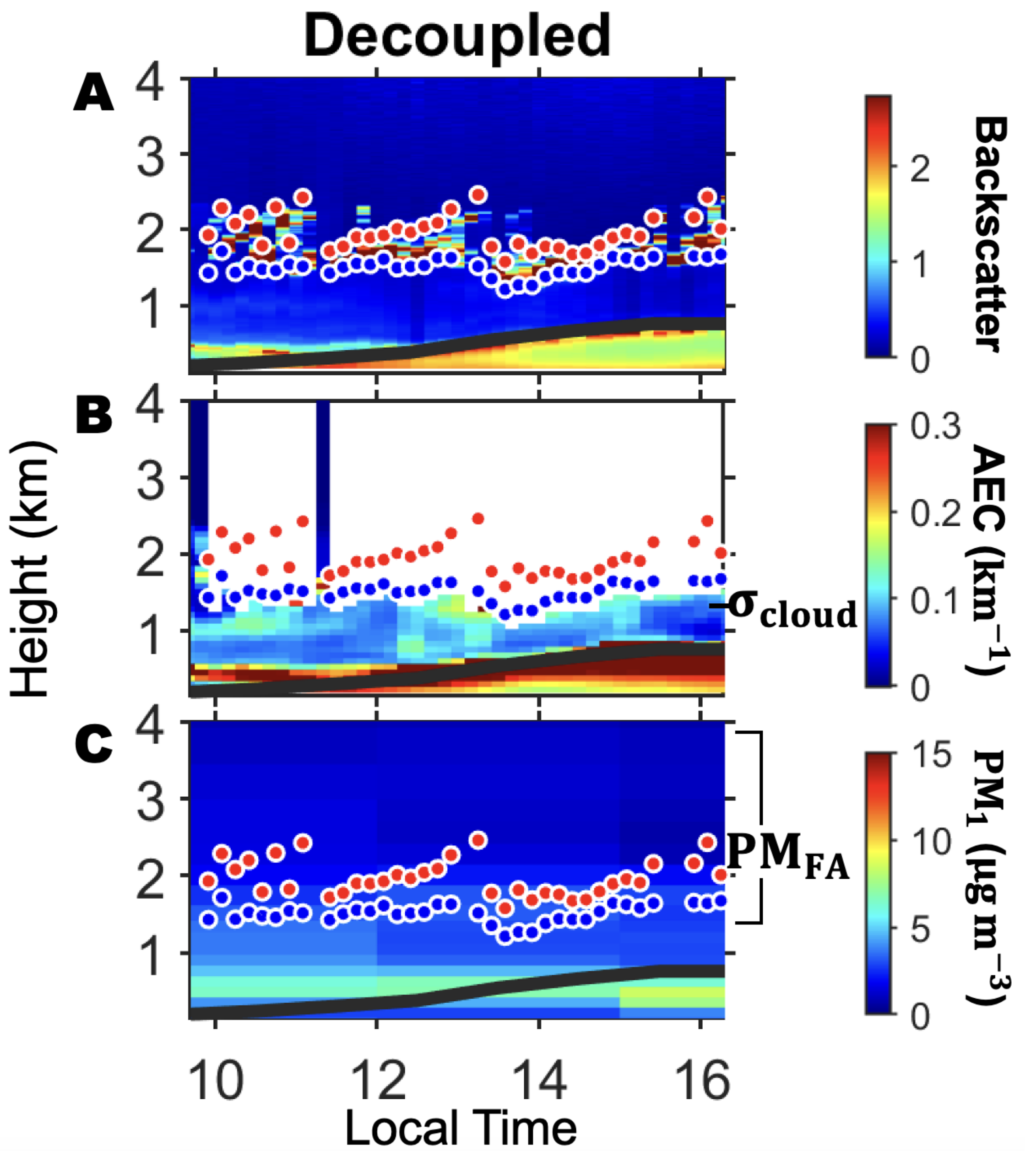

Fig. S3. A decoupled cloud case. (A) Lidar backscatter profiles, (B) Aerosol Extinction Coefficients (AEC) profiles derived from Raman Lidar, and (C) PM_1 profiles obtained from MERRA-2. The cloud base and cloud top are represented by blue and red dots, respectively. The position of the PBL top is marked by a black line. σ_{cloud} indicates the aerosol extinction below the cloud base. PM_{FA} indicates the PM_1 in the free troposphere (FT) and is calculated as the mean value of PM_1 between cloud base and 600 hPa.

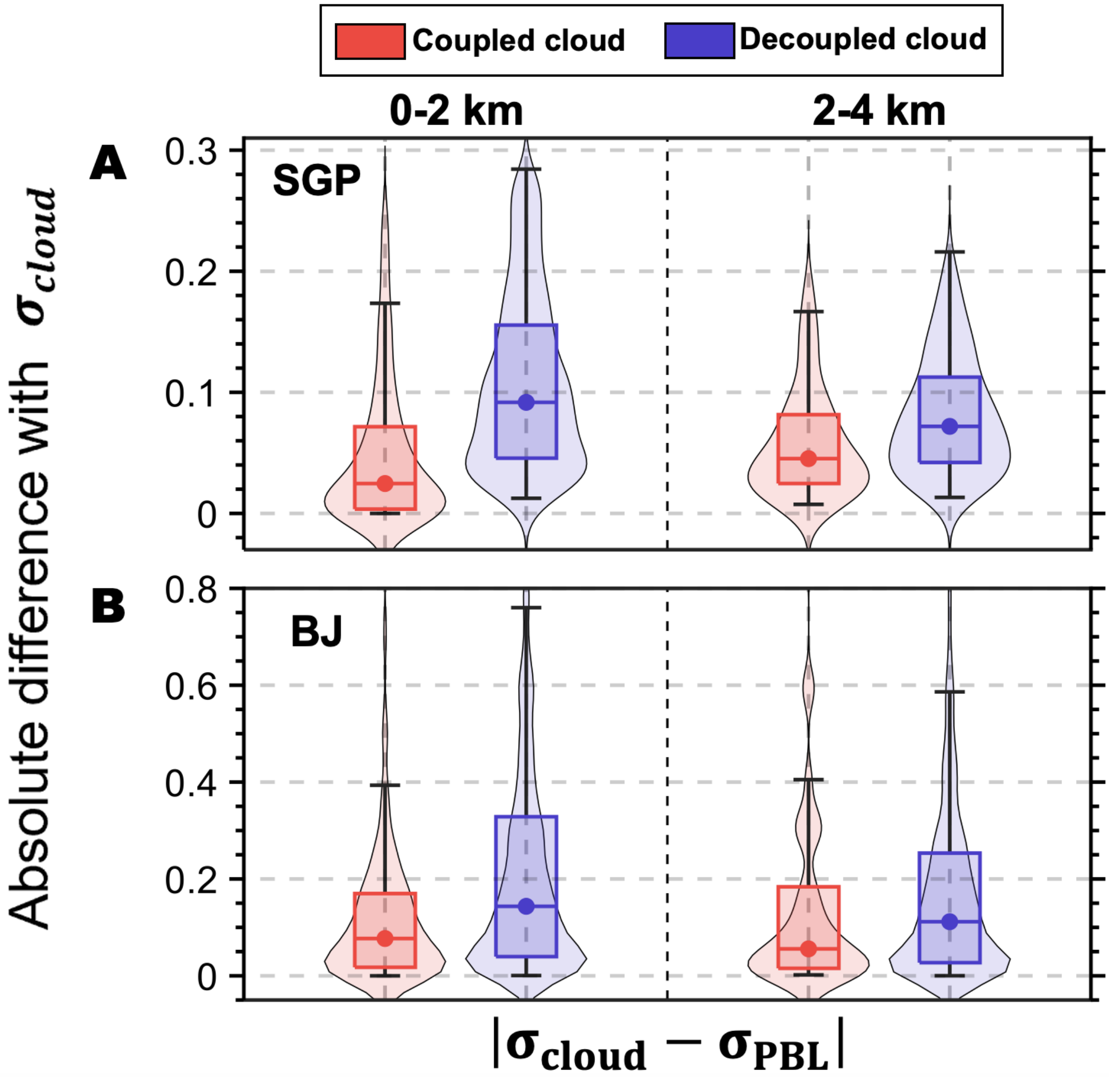

Fig. S4. Sub-cloud aerosol variations under coupled and decoupled regimes. The absolute differences between aerosol extinction (unit: km^{-1}) within boundary layer (σ_{PBL}) and aerosol extinction at the cloud base height (σ_{cloud}) over (A) the SGP site and (B) Beijing. σ_{cloud} is computed as the mean extinction from neighboring clear pixels at the cloud base height. The analysis covers two cloud base ranges: 0-2 km and 2-4 km. Box-and-whisker plots showing the 10th, 25th, 50th, 75th, and 90th percentile values. The width of the color-shaded areas represents the corresponding distribution of absolute differences.

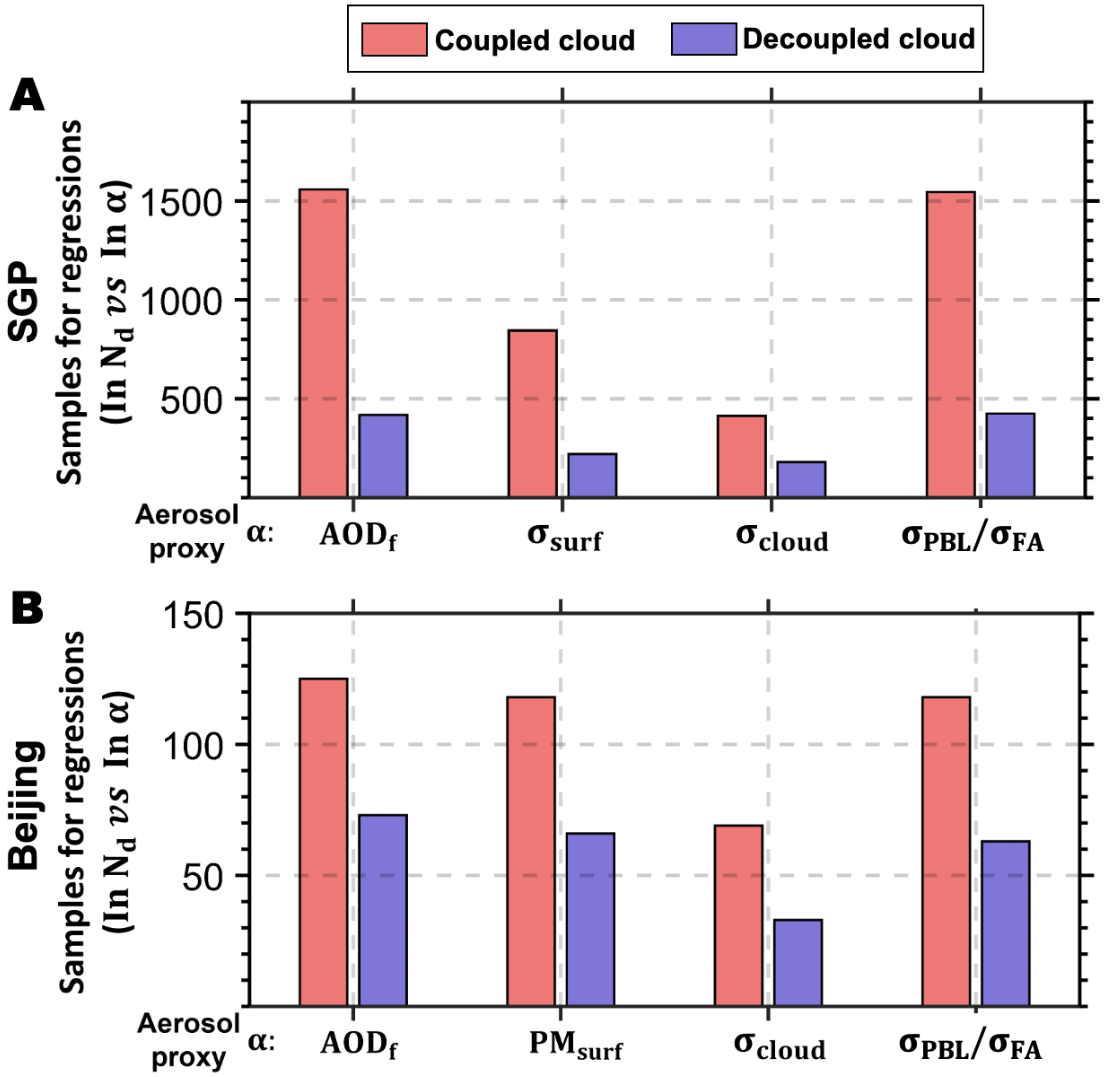

Fig. S5. Number of samples used in regression calculations for both the SGP and Beijing locations. The red bars represent sample counts in hours for the coupled cloud scenario, while the blue bars denote those of the decoupled cloud scenario. Panel (A) showcases samples for SGP, and panel (B) for Beijing. Corresponding regression slopes based on these samples are shown in Figure 2.

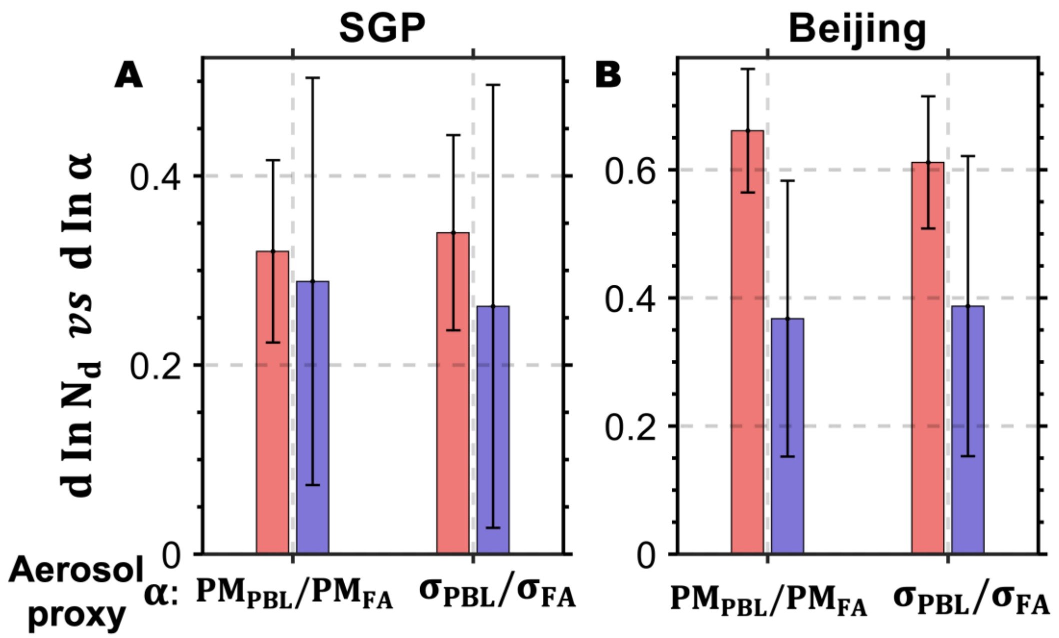

Fig. S6. The responses of cloud droplet number concentration to different aerosol proxies. The responses ($\frac{d\ ln\ N_d}{d\ ln\ \alpha}$) are calculated as the slopes of linear regression between $d\ ln\ N_d$ and $d\ ln\ \alpha$ for liquid water clouds over (A) SGP and (B) Beijing. Red and blue bars indicate the responses for coupled and decoupled cases. The aerosol proxies used are mean PM_1 within PBL (PM_{PBL}) and mean PM_1 within free atmosphere (PM_{FA}), and mean σ_{dry} within PBL (σ_{PBL}) and mean σ_{dry} within free atmosphere (σ_{FA}). The error-bars indicate the 90% confidence level.

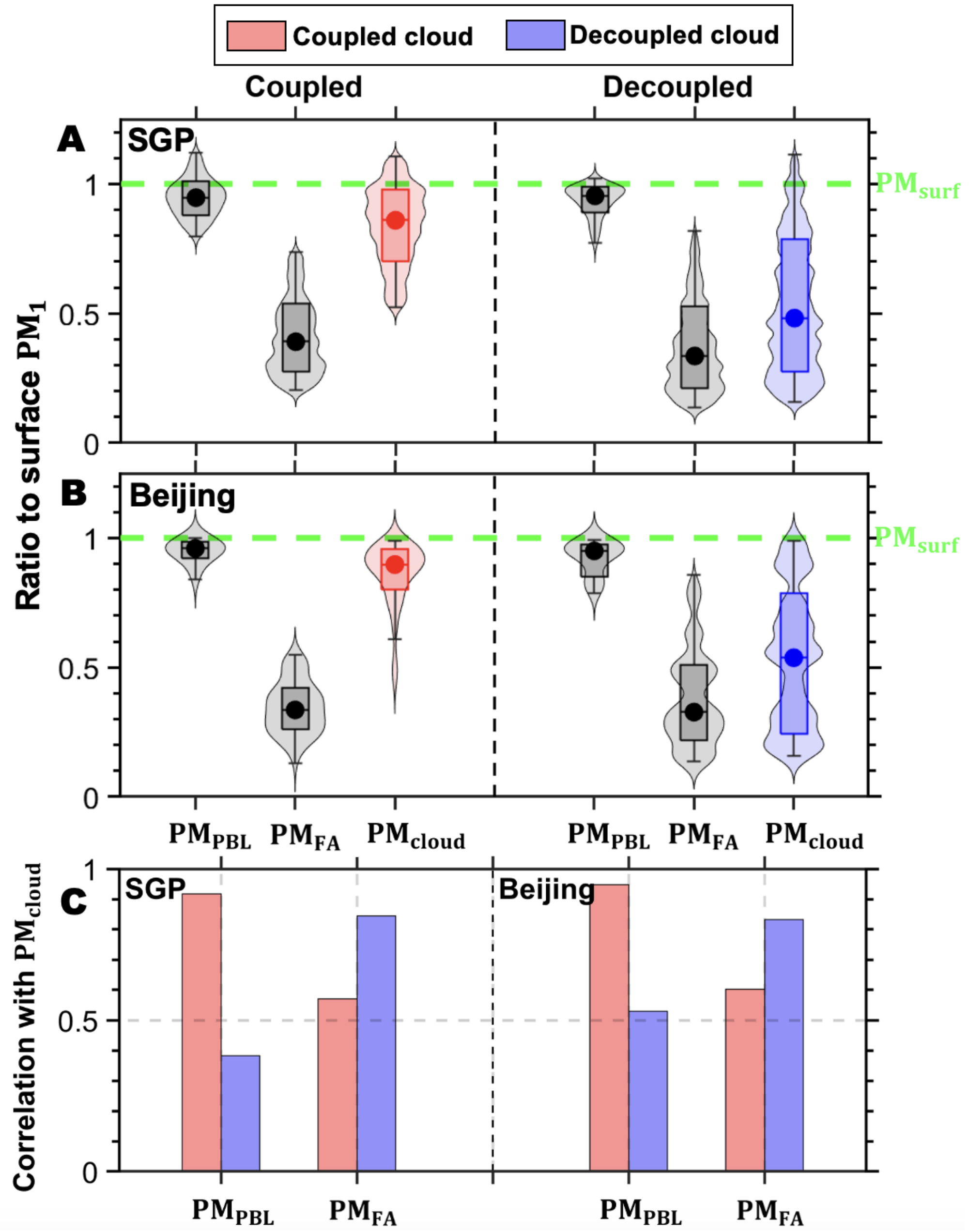

Fig. S7. Values of PM_{PBL} and PM_{FT} over the SGP and Beijing. The ratio between PM_{PBL} and surface PM_1, the ratio between PM_{FA} and surface PM_1, and the ratio between PM_{cloud} and surface PM_1 from MERRA-2 for coupled and decoupled regimes over (A) SGP and (B) Beijing. (C) The correlation coefficients between PM_{cloud} and PM_{PBL}/PM_{FA}.

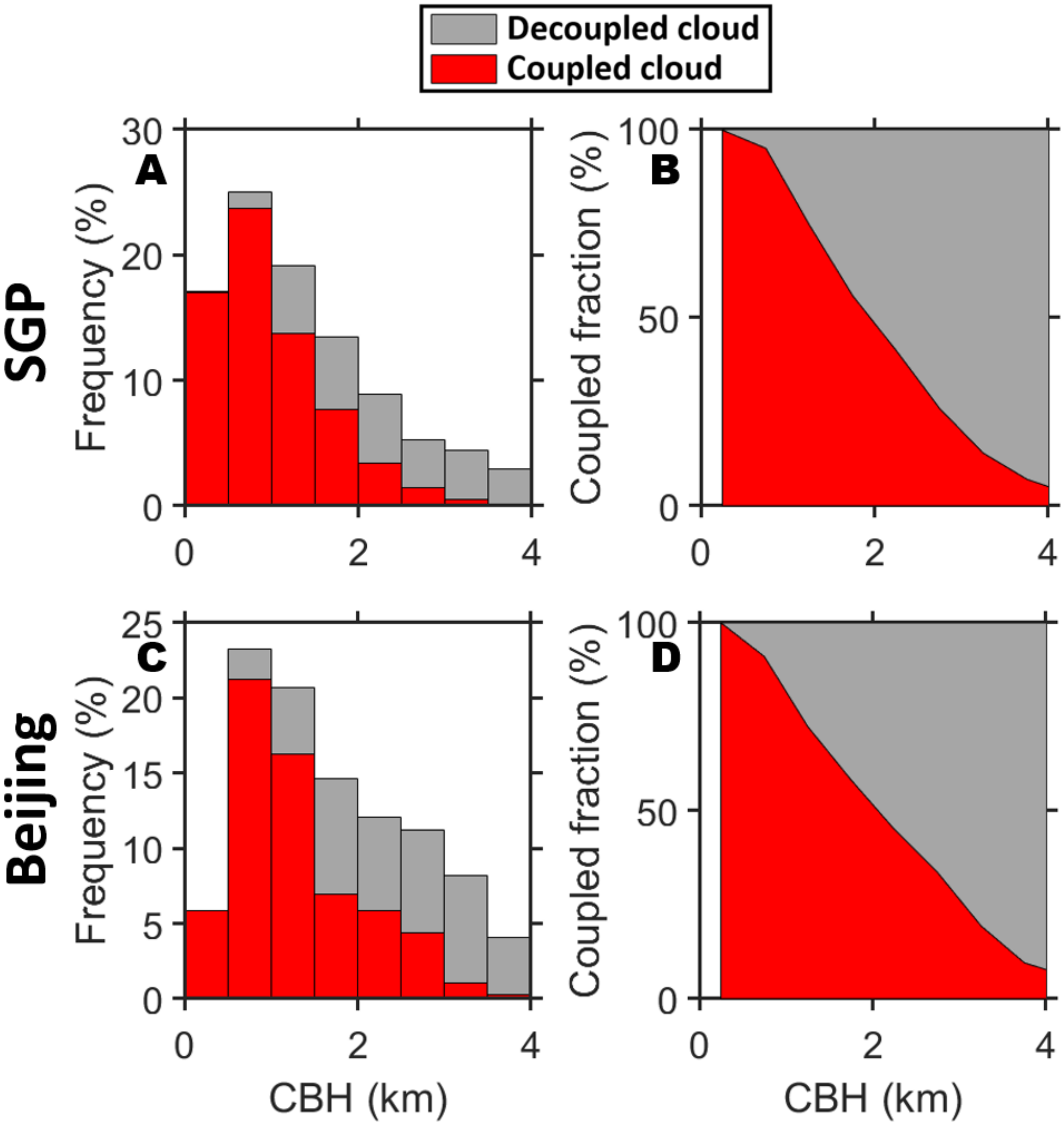

Fig. S8. Climatology and cloud-surface coupling and cloud fraction. The height-dependent occurrence frequencies of the cloud-base height for coupled clouds (red bars) and decoupled clouds (grey bars) over the (A) SGP and (C) Beijing. Panels (B) and (D) represent the coupled fraction (red areas) at different cloud-base heights over the SGP site and Beijing, respectively. Note that only liquid water clouds were considered in this analysis, and instances where coupled and decoupled clouds mixed were excluded.

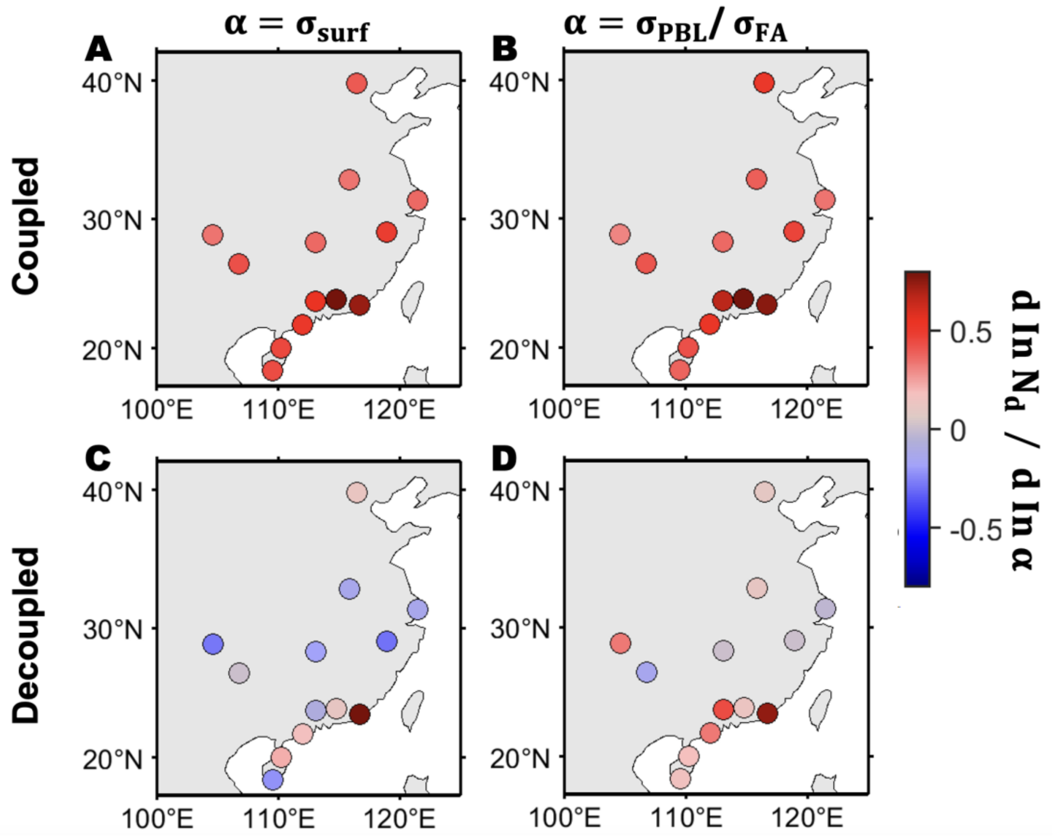

Fig. S9. Responses of cloud droplet number concentration to aerosols. The responses of N_d to aerosols ($\frac{d\ ln\ N_d}{d\ ln\ \alpha}$) is calculated as the slopes of linear regression between $d\ ln\ N_d$ and $d\ ln\ \alpha$ over different radiosonde sites. The aerosol proxies used are surface PM_1 and PM_{PBL}/PM_{FA}. The color shaded dots indicate the value of $\frac{d\ ln\ N_d}{d\ ln\ \alpha}$.

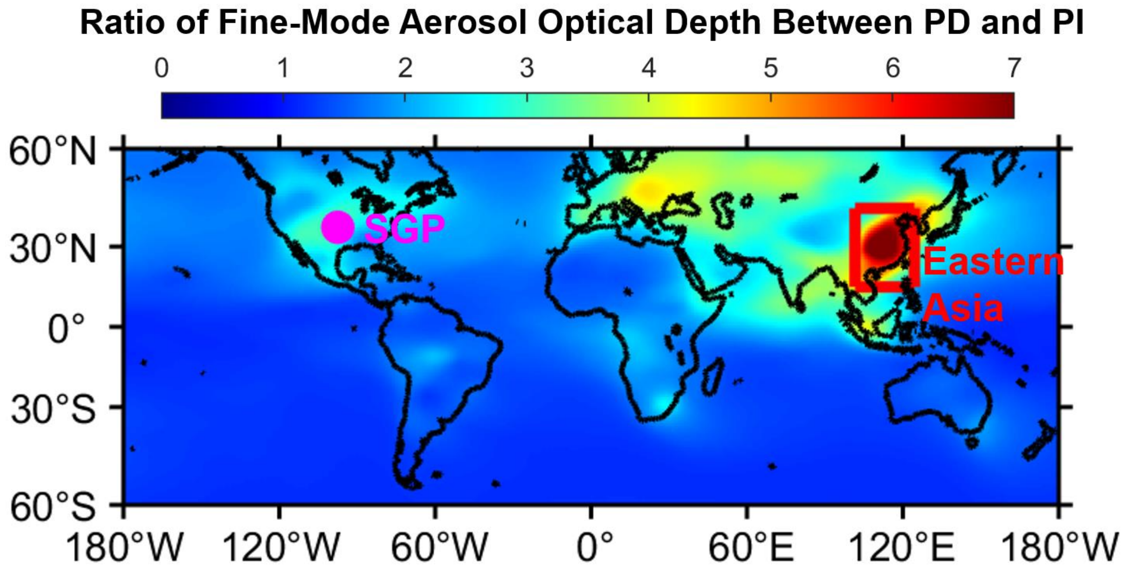

Fig. S10. Comparative Analysis of Fine-Mode Aerosol Optical Depth Between Present Day (2005) and Preindustrial Era (1850). The Fine-Mode Aerosol Optical Depth (AOD_f) for both time periods is derived from the Max Planck Aerosol Climatology version 2 (MACv2). The ratio provides insights into the change in aerosol optical depth from the Preindustrial era to the Present day. Two study regions (SGP and Eastern Asia) have been marked in this Figure.

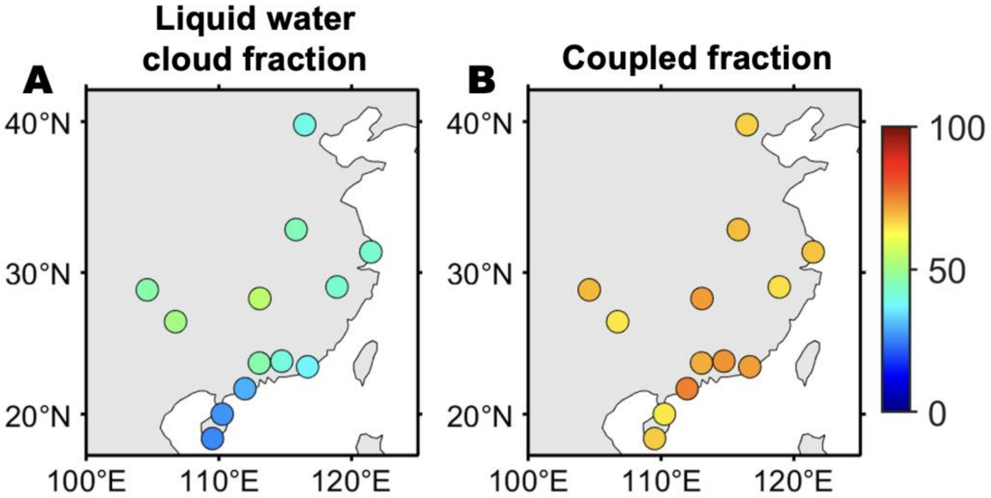

Fig. S11. Cloud fraction and coupled fraction over different radiosonde sites. The color shaded dots indicate the summer mean of (A) liquid water cloud fraction and (B) coupled fraction. The cloud phase is identified by MODIS product, while the cloud-surface coupling is diagnosed by radiosonde profiles.

Table S1. The list of the datasets in the present study, location, periods, and their corresponding description.

Dataset	Location	Period	Description
Beijing Superstation	Beijing, China	July 2017 - October 2019	Observations from a micro-pulse lidar (MPL), surface meteorological instruments, surface PM2.5 observations
Southern Great Plains (SGP)	Oklahoma, USA	October 1998 - December 2020	Measurements of potential temperature, Active Remote Sensing of Clouds, cloud optical properties, radiation budget, and surface aerosol.
In-Situ Aerosol Profiles (IAP) campaign	Oklahoma, USA	2000 - 2006	Dry fine-mode aerosol extinction (diameter<1μm) (σ_{dry}) from the Aerosol Observing System
Radiosonde Stations	Eastern China	Summer 2015 - 2019	Vertical profiles of pressure, water vapor, temperature, and wind at 1400 Local Time.
MODIS Level-2 Cloud	Global	2002-2019	Cloud optical depth, effective radius, top height/temperature, liquid water path, cloud phase, and multilayer flag.
MERRA-2	Global	1998-2020	Re-analyzed AOD_f and vertical profiles of PM_1 at 0.5° x 0.625° resolution.
Max Planck Aerosol Climatology version 2 (MACv2)	Global	N/A	Monthly global maps for aerosol optical properties and compositions with global coverage.
